# Characterization of an HLA-restricted and human cytomegalovirus-specific antibody repertoire with therapeutic potential

**DOI:** 10.1007/s00262-020-02564-1

**Published:** 2020-04-16

**Authors:** Moritz Bewarder, Gerhard Held, Lorenz Thurner, Stephan Stilgenbauer, Sigrun Smola, Klaus-Dieter Preuss, Gabi Carbon, Birgit Bette, Konstantinos Christofyllakis, Joerg Thomas Bittenbring, Arne Felbel, Alexander Hasse, Niels Murawski, Dominic Kaddu-Mulindwa, Frank Neumann

**Affiliations:** 1 Internal Medicine I, Saarland University Medical Center, 66421 Homburg, Germany; 2 José Carreras Center, Saarland University Medical Center, Homburg, Germany; 3 Internal Medicine I, Westpfalz-Klinikum Kaiserslautern, Kaiserslautern, Germany; 4 Institute of Virology, Saarland University Medical Center, Homburg, Germany

**Keywords:** HCMV infection, Immunosuppression, Allogeneic stem cell transplantation, TCR-like antibodies

## Abstract

With an infection rate of 60–90%, the human cytomegalovirus (HCMV) is very common among adults but normally causes no symptoms. When T cell-mediated immunity is compromised, HCMV reactivation can lead to increased morbidity and mortality. HCMV antigens are processed and presented as peptides on the cell surface via HLA I complexes to the T cell receptor (TCR) of T cells. The generation of antibodies against HCMV peptides presented on HLA complexes (TCR-like antibodies) has been described, but is without therapeutic applications to date due to the polygenic and polymorphic nature of HLA genes. We set out to obtain antibodies specific for HLA/HCMV-peptides, covering the majority of HLA alleles present in European populations. Using phage display technology, we selected 10 Fabs, able to bind to HCMV-peptides presented in the 6 different HLA class I alleles A*0101, A*0201, A*2402, B*0702, B*0801 and B*3501. We demonstrate specific binding of all selected Fabs to HLA-typed lymphoblastoid cell lines (EBV-transformed B cells) and lymphocytes loaded with HCMV-peptides. After infection with HCMV, 4/10 tetramerized Fabs restricted to the alleles HLA-A*0101, HLA-A*0201 and HLA-B*0702 showed binding to infected primary fibroblasts. When linked to the pseudomonas exotoxin A, these Fab antibodies induce highly specific cytotoxicity in HLA matched cell lines loaded with HCMV peptides. TCR-like antibody repertoires therefore represent a promising new treatment modality for viral infections and may also have applications in the treatment of cancers.

**Electronic supplementary material:**

The online version of this article (10.1007/s00262-020-02564-1) contains supplementary material, which is available to authorized users.

## Introduction

HCMV is a double-stranded DNA virus and member of the Herpesviridae family. Like all herpesviruses, HCMV persists after acute infection and establishes latent infection in a non- or slowly replicating form. Host cells for latent infection can be neutrophils, T lymphocytes, endothelial cells, renal epithelial cells or salivary glands [2]. Infection with HCMV is very common among adults (60–90%) and primary infection often does not cause any symptoms. In rare cases primary infection can cause HCMV mononucleosis with fever, lymphadenopathy and relative lymphocytosis. Usually, HCMV infection resolves quickly and is controlled by CD8+, CMV-specific T cells [2]. After allogeneic hematopoietic stem cell transplantation (HSCT) or solid organ transplantation, T cell-mediated immunity is often suppressed and HCMV reactivation can significantly contribute to morbidity and mortality after transplantation [3, 4]. HCMV is one of the most common opportunistic pathogens detected after HSCT or solid organ transplantation and can cause severe pneumonia, hepatitis, encephalitis, colitis or ulcers of the gastrointestinal tract [3, 4]. Not only patients undergoing transplantation but also patients with HIV-induced immunodeficiency suffer from HCMV-related diseases like retinitis and polyradiculopathy [5, 6].

Despite serious side effects and the selection of drug-resistant strains, ganciclovir and valganciclovir remain the mainstay in the management of HCMV-associated disease after allogeneic stem cell transplantation and in solid organ recipients [7]. For patients after heart and lung transplantation a universal prophylaxis with (val-) ganciclovir is recommended. For the remaining solid organ transplantations, a preemptive therapeutic strategy guided by detection and surveillance of HCMV DNA or antigen is the standard procedure [3, 8]. The first-line treatment for HCMV disease is usually intravenous (i.v.) ganciclovir or oral valganciclovir. In life threatening cases i. v. ganciclovir in combination with HCMV-specific immunoglobulin should be administered. Second-line therapeutic options are foscarnet and cidofovir [3, 4]. Recently, with the FDA approval of the HCMV terminase inhibitor letermovir, a new class of HCMV drugs for prophylaxis and treatment has become available [9]. But even with letermovir prophylaxis, 38% of patients after allogeneic HSCT developed HCMV infection, illustrating the need for new treatment options of HCMV disease. Another treatment option for HCMV infection after allogeneic HSCT is the transfer of donor-derived HCMV-specific T cells, but this option remains labor-intensive hampering its routine application [10].

The proteome of nucleated cells is displayed permanently on the cell surface by degradation of intracellular proteins and presentation of the peptide fragments in class I major histocompatibility complexes [MHC I; human leukocyte antigen I (HLA I) in humans]. Displaying tumor- or virus-derived antigenic peptides, malignant or infected cells can be distinguished by T cells from their healthy counterparts [11]. HCMV infection is controlled by CD8+ T cells that recognize HCMV peptides presented on HLA class I complexes of infected cells and mount potent immune responses [12]. The humoral immune response plays only a minor role in controlling latent HCMV infection. Long term immunity against HCMV mostly depends on T cells, despite the fact that several antibodies against different HCMV antigens are detectable after primary infection [13].

The identification and generation of antibodies directed against viral peptides presented on HLA I complexes has been described previously. However, so far there are no therapeutic or diagnostic applications of such antibodies, generally referred to as T cell receptor (TCR) like antibodies [14, 15]. The biggest handicap of TCR-like antibodies is their restriction to certain HLA alleles and complexes. To make use of TCR-like antibodies in the diagnosis and treatment of viral infections, a whole repertoire of antibodies covering the HLA variety of a given population would be necessary. Since different peptides can be presented by the same HLA I molecule and an individual expresses a unique set of HLA I alleles, many HLA/HCMV-peptide combinations as targets for TCR-like antibodies are conceivable. The following HLA alleles cover approximately 80% of the German population: HLA-A*0101, HLA-A*0201, HLA-A*2402, HLA-B*0702, HLA-B*0801, HLA-B*3501. Although their frequencies vary, these HLA I alleles are also very common in other European populations. The most immunodominant HCMV-peptides presented by these HLA molecules are derived from the HCMV antigens pp65 and IE-1. Accordingly, they are the most targeted antigens of HCMV by CD8+ T cells [16, 17]. Phage display technology represents a very potent approach in selecting antigen-specific antibodies and can also be used to identify TCR-like antibodies [14, 18].

We set out to obtain antibodies specific for HLA I/HCMV-peptide complexes covering the majority of HLA alleles present in European populations using phage display. Selected Fab antibodies were tested and characterized for specific binding to HLA I/HCMV-peptide complexes by ELISA and flow cytometric analysis using different types of target cells. To show the therapeutic potential of our approach, we incorporated the identified HCMV-specific TCR-like Fabs with pseudomonas exotoxin A (ETA) into immunotoxins to treat HLA matched and HCMV-peptide-loaded cell lines.

## Materials and methods

### Selection of HLA alleles and HCMV-peptides for phage display

To identify the most common HLA class I alleles, we used the online, free accessible “The Allele Frequency Net Database.” For calculation of HLA frequency and distribution, the “Germany pop 8” dataset [19] was used. A comprehensive literature search using the online US national library of medicine of the national institutes of health (Pubmed) was performed to identify HCMV-epitopes that elicit a T cell response [20].

### Generation of HLA I/peptide complexes

HLA I/peptide monomers were produced as previously described [21]. Peptides were bought from GeneCust® (GeneCust, 5690 Ellange, Luxembourg). Plasmids for β2 microglobulin and the HLA A*0201 heavy chain were provided by the Ludwig Institute of Cancer Research (Lausanne Branch, University of Lausanne, Epalinges, Switzerland). Plasmids for A*0101, A*2402 and B*0702 heavy chains were kindly provided by the NIH Tetramer Facility. B*0801 and B*3501 heavy chains were cloned at our laboratory. Heavy chains were biotinylated in vivo using the biotinylation sequence AviTag and an IPTG inducible pASYC vector encoding the BirA enzyme [22].

### Selection of HLA-restricted and HCMV-specific Fabs

The phagemid library used in this study consists of a large, nonimmune human Fab repertoire containing 3.7 × 10^10^ different antibody fragments [23]. 1.8 × 10^12^ phages were pre-incubated in 2% nonfat dry milk, PBS and streptavidin-coated magnetic beads (Hyglos GmbH, 82347 Bernried, Germany). Phages were incubated for 1 h with biotinylated HLA I/peptide complexes at decreasing concentrations (300, 100, 20 and 5 nM). Streptavidin beads were added for 15 min followed by 12 cycles of washing. Bound phages were eluted with 100 mM triethylamine. Sequences of selected antibody clones were evaluated using the “ImMunoGeneTics information system®” and “The National Center for Biotechnology Information” online tools V-QUEST and BLAST [24–26].

### Expression and biotinylation of Fabs and Fab-ETA′ immunotoxins

Recombinant soluble Fab antibodies were expressed in TG1 *E. coli* bacteria and purified by immobilized metal affinity chromatography (IMAC) using Talon beads (Takara Bio USA, Inc., Mountain View, CA, USA) as described previously [27]. In vivo biotinylation was performed as described previously [28]. To generate Fab-ETA′ immunotoxins, the biotinylation sequence AviTag was replaced by the sequence of a truncated version of the pseudomonas exotoxin A (ETA′). For better intracellular transport and efficacy, the 5 C-terminal amino acids of ETA′ were exchanged for the KDEL motif by PCR [29]. Expression of FAB-ETA′ immunotoxins was performed in *E. coli* strain TG1.

### Cell culture

Melanoma cell lines Me 260, Me 275 and SK-mel-23, the squamous cell carcinoma cell line A-431 and the human lung fibroblast cell line MRC-5 were used in this study. All cell lines were cultured in RPMI 1640 medium supplemented with 10% fetal calf serum, 100 units/ml penicillin/streptomycin and 2 mM/ml glutamine. LCLs were generated by in vitro infection of PBMCs with EBV. Peripheral blood was donated from HLA-typed healthy individuals. All cell lines and LCLs were cultured in RPMI 1640 medium supplemented with 10% fetal calf serum, 100 units/ml penicillin/streptomycin and 2 mM/ml glutamine. To generate primary skin fibroblast cell cultures, skin punch biopsies of HLA-typed donors were obtained and treated by standard procedure [30, 31].

### ELISA of phages and Fab antibodies

ELISAs were performed between indirectly coated HLA I/peptide complexes and phage clones or Fab antibodies [32]. Plate-bound streptavidin (5 µg/ml) was incubated with biotinylated HLA I/peptide complexes at 4 µg/ml. To confirm correct folding of HLA I/peptide complexes, we used the conformation-specific monoclonal antibody Tü155 (kindly provided by A. Ziegler, Berlin, Germany). Fab antibodies were incubated with indirectly coated HLA I/peptide complexes at a concentration of 10 µg/ml for 1 h at room temperature. Fab binding was confirmed using the murine anti-myc antibody 9E10 (Roche, Mannheim, Germany) and a horseradish peroxidase-conjugated anti-mouse IgG (Dako, Glostrup, Denmark). Bound phages were detected using the murine IgG antibody M13 (Amersham Pharmacia Biotech, Sweden).

### Generation of Fab tetramers

Soluble Fabs were tetramerized by adding R-phycoerythrin (R-PE) conjugated streptavidin (ProZyme, Ballerup, Denmark) to biotinylated Fab monomers in a molecular ratio of 1:4 [33].

### Peptide loading of LCLs, lymphocytes and cell lines

LCLs, lymphocytes and cell lines were loaded with HCMV- and control-peptides. 5 × 10^5^ LCLs or 100 µl EDTA blood were washed twice with PBS and incubated for 2 h at 37 °C with 20 µg/ml HCMV- or control-peptide. For peptide-loading of cell lines, 5 × 10^3^ cells were washed twice with PBS and incubated over night with 50 µg/ml of either HCMV- or control-peptides.

### Flow cytometry

LCLs or blood cells were incubated for 15 min with biotinylated Fab antibodies (20 µg/ml for LCLs, 50 µg/ml for blood cells) followed by Streptavidin-conjugated R-PE (1:300, 15 min) from Jackson, West Grove, PA, USA. HCMV-infected fibroblasts were detached with trypsin and treated according to LCLs. Fab-tetramers were applied at 20 µg/ml. Fab-ETA′ constructs were detected by anti-Pseudomonas exotoxin A polyclonal rabbit serum (Sigma, St. Louis, Missouri, USA, catalogue number P2318), 1:300 diluted biotinylated anti-Rabbit IgG (DIANOVA GmbH, Hamburg, Germany) and streptavidin-conjugated R-PE (Jackson, West Grove, PA, USA). Experiments were performed on the BD FACS Canto. FACS Diva software and WinMDI 2.8 (Purdue University Cytometry Laboratories) were used for analysis. If possible, at least 10^4^ cells were analyzed. Geometric mean fluorescence intensity (gMFI) values were compared between HCMV-infected and uninfected fibroblasts and given as mean values of all performed experiments.

### Surface plasmon resonance experiments

Surface plasmon resonance imaging was performed with a BIAcore2000 (BIAcore AB, Uppsala, Sweden). Phosphate-buffered saline (pH 7.4) served as running buffer. Capture of Streptavidin (20 µg/ml in 10 mM sodium acetate buffer, pH 4.2) to a CM5 sensor chip was performed using standard amine coupling chemistry to reach a level of 7000 RU. Biotinylated HLA class I/HCMV-peptide complexes were immobilized as ligands at target densities of approximately 150 RU. The purified Fabs were injected at concentrations of 1 µM, 0.5 µM, 0.25 µM, 0.125 µM and 0.0625 µM with a flow rate of 30 µl/min. To establish a baseline, Fabs were injected on a flow cell on which only Streptavidin was immobilized. Association and dissociation rate constants (*k*_a_ and *k*_d_) and the dissociation constant (*KD*) were determined by single cycle kinetics using the BIA evaluation version 4.1.1 software.

### Infection of fibroblast culture with HCMV strain AD169

Cultures of primary fibroblasts and MRC-5 cells were infected at a MOI of 0.5–1.0 with the laboratory HCMV strain AD169, which was kindly provided by Prof. S. Smola.

### Assessment of cell viability

Cells were loaded with 10–50 µg/ml HCMV- or control peptide at 37 °C overnight. Anti-Pseudomonas exotoxin A polyclonal rabbit serum (Sigma, St. Louis, Missouri, USA, catalogue number P2318) was used to show binding of Fab-ETA′ immunotoxins to peptide loaded cell lines. 5 × 10^3^ peptide loaded cells per well were seeded in 96-well plates (Nunc) and incubated with Fab-ETA′ constructs (0.1–15 µg/ml) for 24–48 h. 10 µl of alamarBlue™ (Catalogue number DAL1025, from Invitrogen, Carlsbad, CA, USA) [34] were added into each well for a total volume of 100 µl. Data were collected using an Infinite® 200 PRO microplate reader from Tecan (Männedorf, Swiss). All experiments were performed at least in triplicate. Changes in viability were detected as reduction in fluorescence. Estimated viability of cell lines after treatment is given as relative viability.

## Results

### Identification of suitable HLA I/HCMV-peptide complexes

Using the allele frequency net database [19], we identified the 7 most prevalent HLA I alleles in the German and most European populations. We found that T cell responses with specificity for 6 HCMV-derived peptides presented on 6/7 of these most prevalent HLA I complexes, have previously been described [35–38]. These 6 HLA I alleles (Table 1) occur in up to 80% of many European populations [39]. Five of six peptides that induce T cell responses derive from the immunodominant HCMV-antigen pp65 and one peptide derives from IE1 (Table 1). We used these 6 HLA I/HCMV-peptide complexes to select and characterize TCR-like, CMV-specific antibodies by phage display.

**Table 1:** HLA alleles, their distribution, HCMV-derived T cell epitopes, their HLA-restriction and corresponding binding scores

HLA allele	A*0101	A*0201	A*2402	B*0702	B*0801	B*3501
Allele frequency (German reference)	15.1%	26.7%	9.5%	12.0%	9.5%	6.2%
HCMV antigen	pp65	pp65	pp65	pp65	IE1	pp65
Antigen-derived peptide	YSEHPTFTSQY	NLVPMVATV	QYDPVAALF	TPRVTGGGAM	ELRRKMMYM	IPSINVHHY
SYFPEITHI score	29 (predicted for HLA A*01)	30	24	19	24 (predicted for HLA B*08)	20

### Selection of HLA I/HCMV-peptide-specific Fabs

A total of 10 Fabs were obtained covering 6 HLA I alleles. Two different HLA I-restricted, HCMV-specific Fab clones could be obtained for the alleles A*0101 and B*0702, three different Fabs were identified for the HLA allele A*0201 and only one HCMV-specific clone for the remaining alleles. Further details regarding the sequences and variable regions of the Fabs are given in the supplement (Tables S1 and S2).

### Characterization of the HLA I/HCMV-peptide-specific Fabs

#### TCR-like Fabs tested by ELISA

**Fig. 1:**
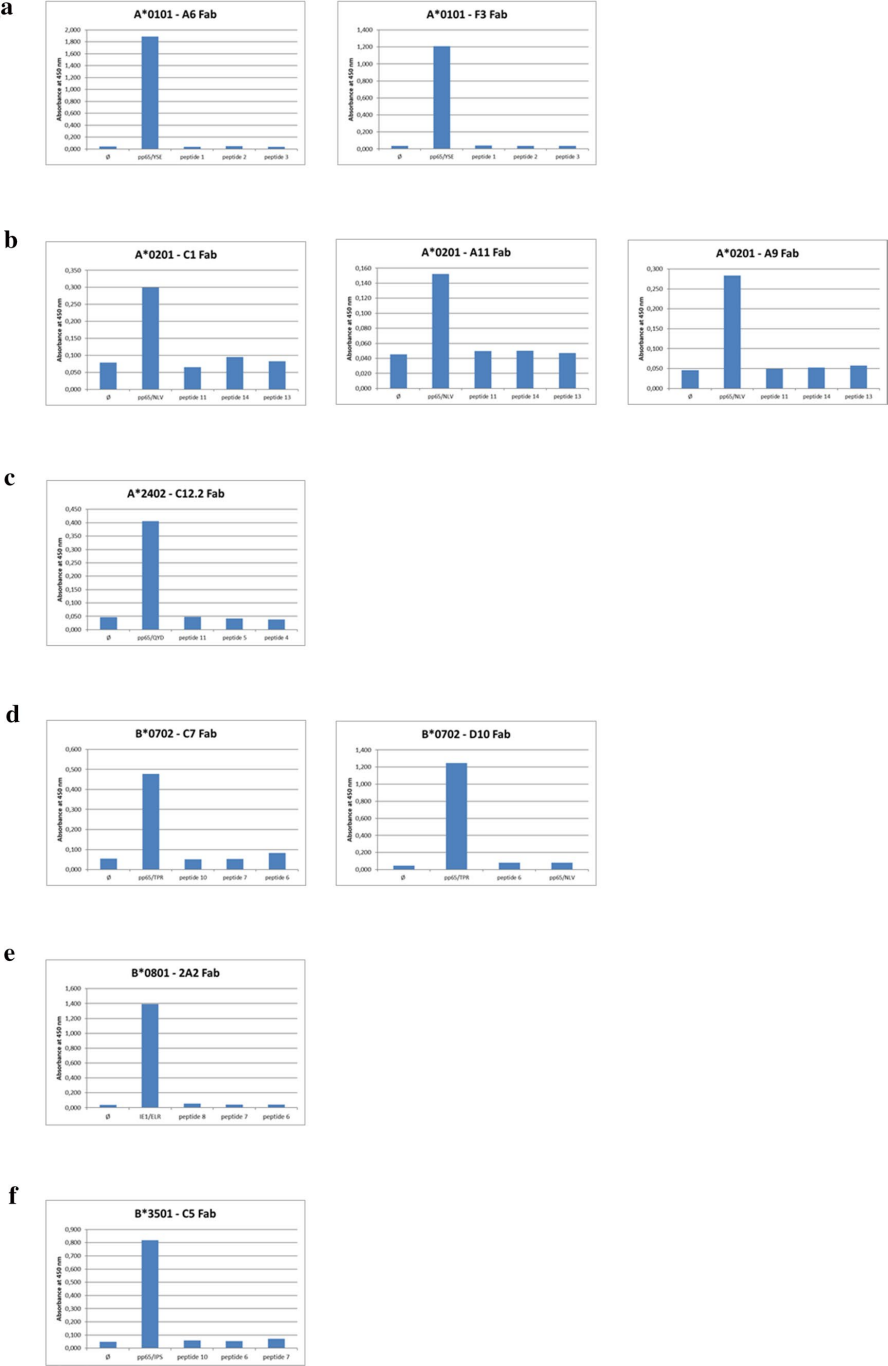
Specificity of selected HLA I/HCMV-peptide-specific Fabs as measured by ELISA. **a–f** show ELISA data of all identified HLA-restricted and HCMV-peptide-specific Fab antibodies sorted by HLA restriction. Biotinylated HLA I complexes presenting a matching HCMV peptide (Table 1) or 3 control-peptides of different origins (Table S3) were folded and indirectly coated onto ELISA plates at a concentration of 4 µg/ml via streptavidin (5 µg/ml). HLA-restricted and HCMV-specific Fabs were applied at a concentration of 10 µg/ml. HCMV target peptides used for HLA I complex folding are shown in Fig. 1 beneath the columns (abbreviated to first 3 amino acids) with the originating HCMV antigen. **a** Shows the A*0101 restricted, HCMV-peptide-specific Fabs A6 and F3. Three Fabs (C1, A11, A9) could be identified, that are specific for HCMV-peptides presented in the A*0201 HLA allele (**b**). Only one Fab with specificity for HLA I/HCMV-peptides was selected for the HLA alleles A*2402 (C12/2 Fab in **c**), B*0801 (2A2 Fab in **e**) and B*3501 (C5 Fab in **f**). The Fabs C7 and D10 show binding capacity to HCMV-peptides but not to control peptides presented in the MHC complex of the B*0702 allele (**d**)

We used ELISAs to test for unselective binding of selected Fabs to corresponding HLA I complexes folded with different control peptides (Table S3). HLA I/HCMV-peptide complexes and HLA I/control-peptide complexes were coated on ELISA microplates. All described Fabs exclusively bound to their matching HLA I/HCMV-peptide complex but not to any control-peptide presented by the same HLA complex (Fig. 1).

#### TCR-like Fabs on peptide-loaded LCLs and lymphocytes

**Fig. 2:**
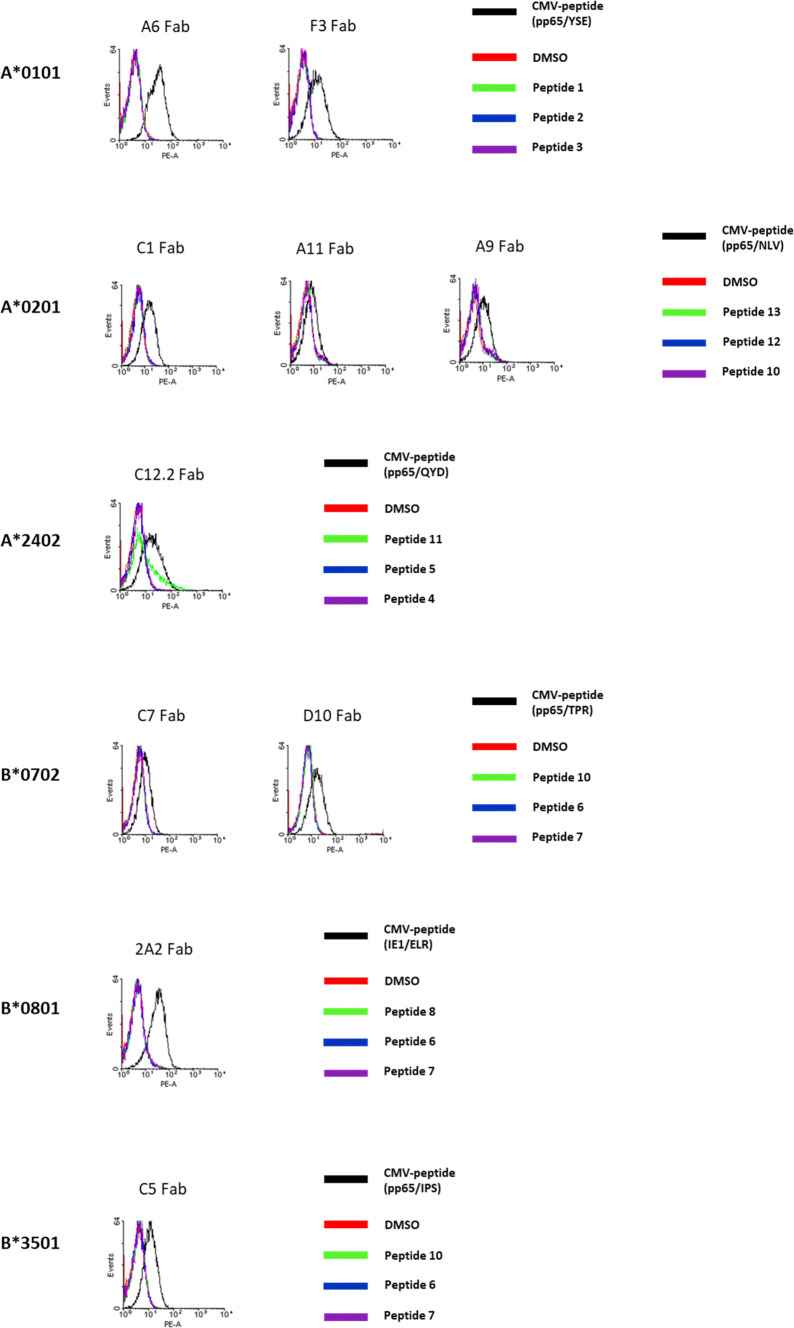
Binding assay of HLA I/HCMV-peptide-specific Fabs on LCLs. LCLs were generated by EBV infection of lymphocytes of HLA-typed donors. Peptide pulsing was done by incubation with 20 µg/ml HCMV-peptide (black line) and three control peptides (green, blue and purple lines) for 2 h at 37 °C. HCMV-peptides used for LCL pulsing are listed in Table 1 and control peptides are listed in Table S3. For staining experiments, peptide-pulsed LCL cells were incubated with 20 µg/ml biotinylated HLA I/HCMV-peptide-specific Fab antibodies for 15 min at RT. Histograms of the staining experiments are assorted from top to bottom by HLA I alleles. A*0101: HLA-restricted and HCMV-specific Fabs A6 and F3 were tested and showed specific binding to HCMV-peptide-loaded LCLs expressing the allele A*0101. Control peptides used were peptides 1, 2 and 3. A*0201: The three TCR-like Fab antibodies C1, A11 and A9 bind to HCMV-peptide-pulsed LCLs and not to the same LCLs pulsed with the control peptides 13, 12 and 10. A*2402: Binding assay of the HLA I/HCMV-specific Fab C12.2 (control peptides 11, 5 and 4) showing specific binding. B*0702: Histograms of C7 and D10 after incubation with HLA matched LCLs. Both Fabs interact only with LCLs that were pulsed with HCMV-peptide and not with same LCLs pulsed with the control peptides 10, 6 and 7. B*0801: 2A2 binds to HCMV-peptide-pulsed LCLs. As controls, the peptides 8, 6 and 7 were used. B*3501: The Fab antibody C5 shows affinity towards HCMV-peptide-pulsed LCLs (control-peptides used were 10, 6 and 7)

LCLs were loaded with HLA-matching HCMV-peptide or with control-peptides for further testing of the TCR-like Fab antibodies by flow cytometry. All Fabs proved to bind specifically to HCMV-peptide loaded LCLs expressing the corresponding HLA allele and did not bind to same LCLs loaded with control-peptides or DMSO mock-loaded LCLs (Fig. 2). Moreover, all Fabs showed no unspecific binding to LCLs with not-matching HLA alleles (*data not shown*).

To determine detection limits of the selected TCR-like Fabs we performed experiments titrating HCMV-peptides and Fab antibodies. LCLs were loaded with matching HCMV-peptides at constant concentrations of 20 µg/ml and stained with TCR-like Fab antibodies in decreasing concentrations from 20 to 0 µg/ml (Figure S1). At a concentration of 20 µg/ml, all Fabs demonstrated clear binding to HCMV-peptide-pulsed LCLs, with some of the Fabs showing binding capacity down to concentrations of below 1 µg/ml. Reversely, the concentrations of HCMV-peptides used for LCL-loading were titrated from 20 to 0 µg/ml (Figure S2). Fab staining of HCMV-peptide-pulsed LCLs was possible down to HCMV-peptide concentrations of 2.5 µg/ml.

HCMV-specific Fab antibodies were further tested on HCMV-peptide-loaded native lymphocytes. HLA A*0101, A*0201 and B*0701 positive lymphocytes were isolated from the same donors LCLs were generated from. HLA A*2402, B*0801 and B*3501 expressing lymphocytes were obtained from different donors as the HLA-corresponding LCLs. Staining of lymphocytes with HLA I/HCMV-peptide-specific Fabs showed similar results as the LCL experiments (Figure S7). In order to exclude relevant interpatient variability, we performed staining experiments of peptide-loaded lymphocytes from different donors (Figure S4). For most Fabs, some difference in binding affinity between two lymphocyte donors was detected, but their general ability to bind to HCMV-peptide-loaded lymphocytes of matching HLA I-status was not affected and therefore not donor-dependent.

#### TCR-like Fabs and Fab tetramers on HCMV-infected fibroblasts

To evaluate the ability of the Fab antibodies to recognize naturally processed HCMV-peptides, we used HCMV-infected primary skin fibroblasts obtained from HLA I-typed volunteers. We established 11 primary skin fibroblast cultures (Table S4). Infection with the HCMV strain AD169 was confirmed by western blot analysis of the HCMV-antigen pp65 (*data not shown*). Monomeric Fabs showed no binding to HCMV-infected fibroblasts as measured by flow cytometry (*data not shown*). To enhance staining intensity, we generated Fab tetramers and assembled seven HCMV specific Fab tetramers from following Fabs: ***A6***, ***F3***, ***C1***, ***C12.2***, ***C7***, ***2A2*** and ***C5 ***(Table 2).

**Table 2:** HLA I/HCMV-specific Fabs obtained by phage display

HLA allele	A*0101	A*0201	A*2402	B*0702	B*0801	B*3501
ID Fab #1	A6	C1	C12/2	C7	2A2	C5
ID Fab #2	F3	A11	–	D10	–	–
ID Fab #3	–	A9	–	–	–	–

**Fig. 3:**
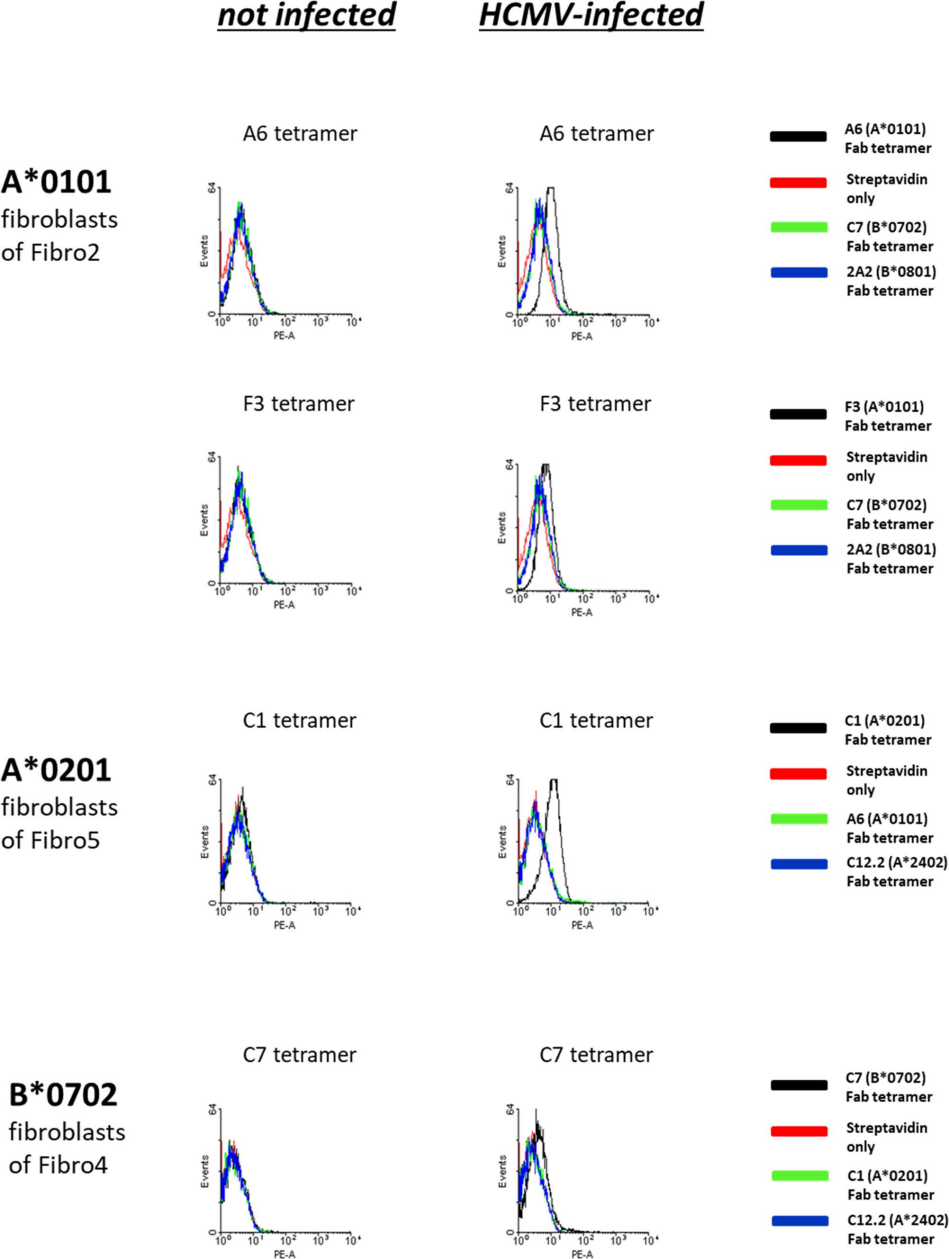
Binding assays of HLA I/HCMV-peptide-specific Fab tetramers to HCMV-infected fibroblasts. Tetramerized HCMV-specific Fab antibodies restricted to the HLA alleles A*0101, A*0201 and B*0702 show binding only to HCMV infected fibroblasts expressing the respective HLA allele and not to uninfected primary human skin fibroblasts (histograms on the left). All generated primary fibroblast cell cultures are shown in Table S4. A*0101: Both Fabs A6 and F3 show binding capacity to the HCMV infected primary fibroblast cells of Fibro2. As negative controls infected as well as uninfected fibroblasts were incubated with the Fabs C7 and 2A2 which are HCMV-specific but restricted to the HLA alleles B*0702 and B*0801 not expressed by Fibro2. A*0201: Tetramerized Fab C1 bound specifically to HCMV infected fibroblasts of Fibro5. As negative controls fibroblasts were incubated with the A*0101 and A*2402 restricted Fabs A6 and C12.2. B*0702: The C7 Fab tetramer and not the tetramers of C1 and C12.2, which are HCMV specific but restricted to the HLA I alleles A*0201 and A*2402, bound to the HCMV infected primary skin fibroblast cell culture Fibro4. None of the Fabs bound to uninfected fibroblasts of Fibro4

As shown in Fig. 3, Fab tetramers of the Fabs ***A6***, ***F3***, ***C1*** and ***C7*** bound to HCMV-infected, HLA-matched fibroblasts. Not-infected fibroblasts or HCMV-infected fibroblasts expressing different HLA alleles served as controls and could not be stained. Fab tetramer staining experiments were repeated at least 4 times on different fibroblast cells (Figure S3). P-values of the difference in fluorescence intensity with and without HCMV-infection were 0.030, 0.0001 and 0.014 for ***A6***-, ***C1***- and ***C7***-tetramers, respectively, demonstrating statistically significant binding of Fab tetramers to HCMV-infected fibroblasts. ***C12.2***, ***2A2*** and ***C5*** (Table 2) tetramers did not bind HCMV-infected fibroblasts with permissive HLA alleles (Figure S8).

### Determination of binding kinetics and affinity of ***A6***, ***C1*** and ***C7***

Using surface plasmon resonance, we determined binding kinetics and affinity of the HCMV specific, TCR-like Fab antibodies ***A6***, ***C1*** and ***C7*** to HLA I/HCMV-peptide complexes (Figure S9). For the Fabs ***A6***, ***C1*** and ***C7 ***dissociation constant (KD) values of 7.6e10^−9^, 6.6e10^−7^ and 1.9e10^−6^ were calculated, showing the highest affinity for ***A6***. Association rate constants (*k*_a_) for ***A6***, ***C1*** and ***C7 ***were 7.78e10^4^, 4.63e10^4^ and 1.01e10^4^, respectively. Dissociation rate constants (*k*_d_) were 5.89e10^−4^ (***A6***), 2.99e10^−2^ (***C1***) and 1.94e10^−2^ (***C7***) (Table S5).

### Cytotoxic effects of HLA I/HCMV-peptide-specific immunotoxins

**Fig. 4:**
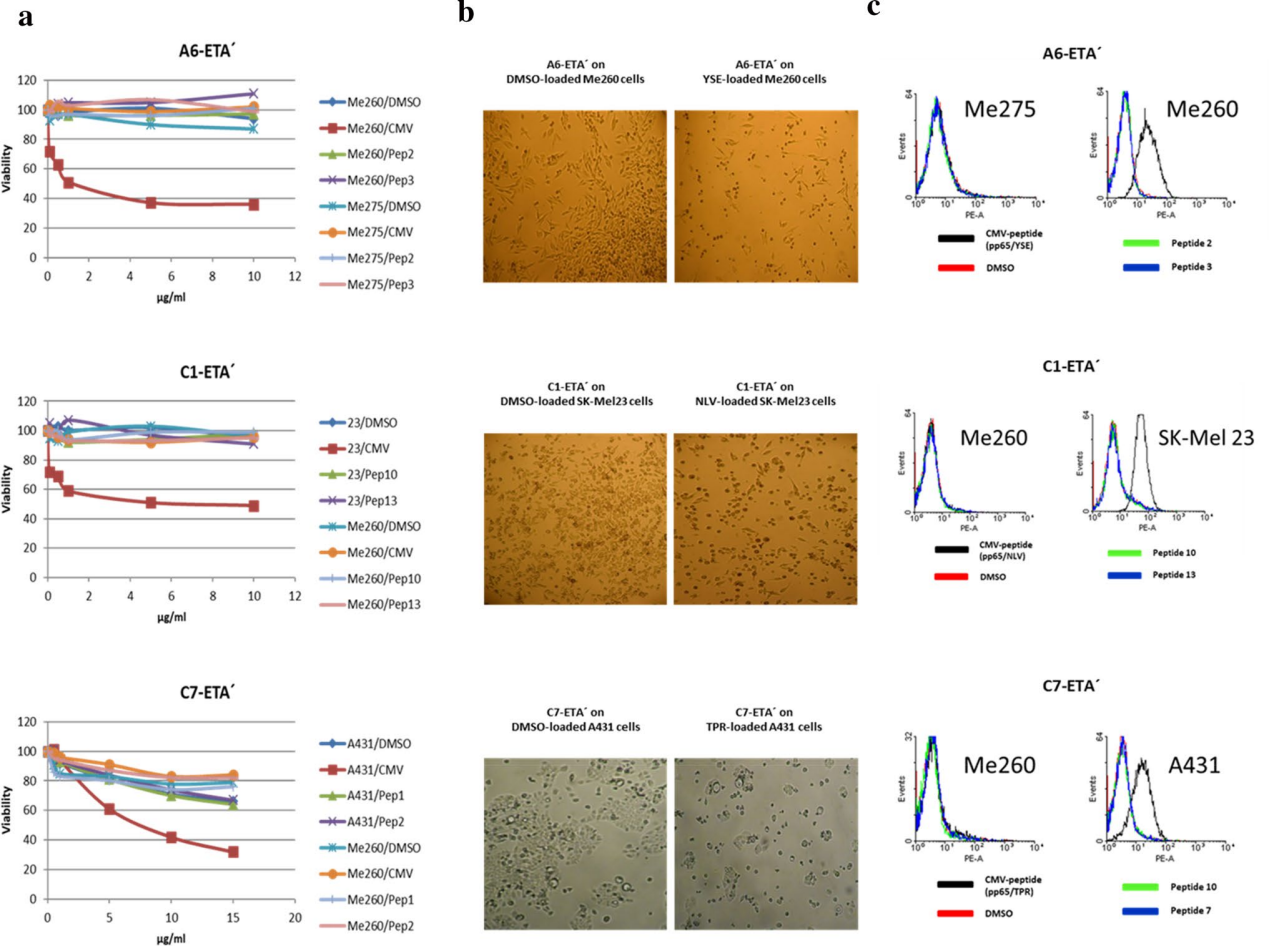
Highly specific cytotoxic effects of A1, C1 and C7 on HLA-matched cell lines loaded with HCMV peptides. To test the therapeutic potential of the identified HCMV-specific and HLA-restricted Fab antibodies, we generated TCR-like immunotoxins linking respective Fabs to a truncated version of the pseudomonas exotoxin A (ETA′). Cytotoxicity was assessed using alamarBlue® cell viability assays after 24–48 h of incubation with the generated immunotoxins at different concentrations ranging from 0.1 to 15 µg/ml. **a***A6-ETA*′: As shown in the top diagram A6-ETA′ was highly specific in killing A*0101 expressing melanoma cells of the cell line Me 260 that were loaded with the appropriate HCMV peptide *pp65/YSE* (*Me260/CMV*). When loaded with DMSO alone or control peptides (Table S3), the viability of these cells was not affected (*Me260/DMSO*, *Me260/Pep2*, *Me260/Pep3*). Cells from a cell line expressing a different HLA allele than A*0101 pulsed correspondingly with HCMV (*pp65/YSE*)- and control peptides were also not affected (*Me275/DMSO*, *Me275/CMV*, *Me275/Pep2*, *Me275/Pep3*). *C1-ETA*′: We tested C1-ETA′ on cells of the A*0201 positive cell line SK-mel-23 that were either mock-loaded with DMSO or matching HCMV (*pp65/NLV*)- and control peptides (*23/DMSO*, *23/CMV*, *23/Pep10*, *23/Pep13*). As further controls, we used cells of the A*0201 negative cell line Me 260 that were mock-loaded with DMSO or pulsed with matching HCMV- and control peptides (*Me260/DMSO*, *Me260/CMV*, *Me260/Pep10*, *Me260/Pep13*). HCMV-peptide-loaded SK-mel-23 cells that were treated with C1-ETA′ showed reduced viability of 50% as compared to controls. *C7-ETA*′: This HCMV-specific TCR-like immunotoxin was tested on cells of the B*0702 expressing cell line A431 and the control cell line Me 260 which does not express B*0702. As seen with the other immunotoxins, C7-ETA′ exerted cytotoxic effects specifically on B*0702 positive cells pulsed with the respective HCMV-peptide (*pp65/TPR*). All cells were either mock-loaded with DMSO or matching HCMV- and control peptides (*A431/DMSO*, *A431/CMV*, *A431/Pep1*, *A431/Pep2*, *Me260/DMSO*, *Me260/CMV*, *Me260/Pep1*, *Me260/Pep2*). **b** Photographs of Me 260, Sk-Mel 23 and A431 cells after treatment with Fab-ETA′ constructs (10–15 µg/ml). Images on the left show cells mock-loaded with DMSO. Images on the right show cells loaded with matching HCMV-peptides. HCMV-peptides and Fab-ETA′ constructs are indicated in the heading. **c** Flow cytometric binding assays of Fab-ETA′ constructs to peptide loaded (50 µg/ml HCMV- or control-peptide) cell lines used for cytotoxicity assays. A6, C1 and C7 ETA′ constructs (25 µg/ml) showed specific binding to HCMV-peptide loaded cell lines expressing matching HLA alleles, whereas HLA matching cell lines loaded with control-peptides could not be stained. Also, cell lines of not matching HLA I status that were loaded with HCMV-peptides showed no binding to respective Fab-ETA′ constructs

***A6***, ***C1*** and ***C7*** were linked to a truncated version of pseudomonas exotoxin A (ETA′) in order to show the therapeutic potential of HCMV specific, TCR-like Fab antibodies (Fig. 4). ***A6-ETA***′ showed highly specific killing of A*0101 expressing Me 260 cells loaded with HCMV peptide. Relative viability of these cells was reduced to 40% as compared to Me 260 cells loaded with control peptides or HCMV-peptide-loaded Me 275cells (A*0101-negative). ***C1-ETA***′ reduced the relative viability of HCMV-peptide-loaded A*0201-positive SK-mel-23 cells to 50% as compared to controls (SK-mel-23 cells loaded with control-peptides, HCMV-peptide-loaded A*0201-negative Me 260 cells). ***C7-ETA***′ was tested on B*0702-positive A431 cells and B*0702-negative Me 260 cells as control. As seen with the other immunotoxins, ***C7-ETA***′ treatment resulted in a reduction of cell viability to 30% specifically in A431 cells pulsed with HCMV-peptide. At ***C7-ETA***′ concentrations above 5 µg/ml, the immunotoxin showed unspecific cytotoxic effects as it reduced the relative viability of the controls to 60–80%.

### C1-ETA′ on HCMV-peptide-loaded MRC-5 cells

MRC-5 cells are HLA A*0201 expressing fibroblasts that can be infected with the HCMV strain AD169. After loading with HCMV-peptide, ***C1-ETA***′ shows specific binding to MRC-5 cells (see figure S5a). After IFNγ treatment, ***C1-ETA***′ educed the relative viability of MRC-5 cells pulsed with 50 µg/ml HCMV-peptide to less than 40% (see figure S5 c). To determine the amount of HCMV-peptide presented on the surface of HCMV-infected MRC-5 cells, we performed HCMV-peptide titration experiments and found comparable staining intensities for MRC-5 cells loaded with 10–20 µg/ml HCMV-peptide as for HCMV-infected MRC-5 cells (see figure S6a and b). When incubated with MRC-5 cells loaded with 12.5 µg/ml HCMV-peptide, ***C1-ETA***′ was able to reduce MRC-5 cell viability, demonstrating its ability to be effective even when the target peptide is presented only in low concentrations (Figure S6 c).

## Discussion

In situations where immunosuppression is mandatory, HCMV-specific TCR-like antibodies may help to overcome HCMV infections. The major limitation of TCR-like antibodies is their restriction to a single HLA I allele [40] and almost all TCR-like antibodies that have been described so far are restricted to the HLA allele families A*02, A*24 and A*01 [14, 41]. To make use of TCR-like antibodies in the treatment of HCMV infections, a whole TCR-like antibody repertoire covering more HLA I alleles and their respective HCMV-peptides is necessary.

Here, we describe an HCMV-specific, TCR-like antibody repertoire which is restricted to 6 HLA alleles that are highly prevalent in most European populations. On LCLs and lymphocytes that were externally loaded with HCMV-peptide, the selected TCR-like Fab antibodies showed specific binding. We tried to determine the detection limits of each selected TCR-like Fab antibody by performing titration experiments of the HCMV-peptide concentration used for LCL loading as well as of the Fab antibody concentration. Both varied significantly between individual TCR-like Fabs and the lower detection limit for HCMV-peptide-loading concentrations was found to be at 2.5 µg/ml.

In an experimental setting simulating natural HCMV infection more adequately, Fabs were tested on HCMV-infected primary fibroblasts. Since presentation of naturally processed peptides resulting from infection is weaker compared to peptide loading, we repeated the staining experiments using tetramerized Fabs to increase their avidity. In doing so, we demonstrated binding to HCMV-infected primary fibroblasts for 4/10 of the HLA I/HCMV-peptide-specific Fab antibodies selected in this study. Due to the fact that these HMCV-specific TCR-like Fab antibodies are restricted to the highly prevalent HLA alleles A*0101, A*0201 and B*0702, up to 50% of European patients would be eligible for treatment with respective TCR-like Fabs. Interestingly, not all HLA/HCMV-peptide-specific Fabs that tested positive on HCMV-peptide-pulsed LCLs and lymphocytes showed also binding to HCMV-infected fibroblasts. We attribute this discrepancy to immune evasion mechanisms of HCMV that are of no relevance after peptide pulsing and to the different affinities of selected TCR-like Fabs. The amount of HLA complexes and of HCMV-peptide on the cell surface of infected fibroblasts will be much lower after infection than after peptide-loading.

To show the therapeutic potential of the identified HCMV-specific, TCR-like Fab antibodies we determined the binding affinity and cytotoxic efficacy of ***A6***, ***C1*** and ***C7***, which are restricted to the most prevalent HLA class I alleles A*0101, A*0201 and B*0702. We first used ETA′-coupled immunotoxins of ***A6***, ***C1*** and ***C7 ***on HLA-matched infected fibroblasts but could not detect any cytotoxic effects, which was attributed to a lack of internalization of HLA/peptide complexes by fibroblasts. Since HCMV infection of the alternatively used cell lines Me260, SK-mel-23 and A431 was not possible, external HCMV-peptide loading was used as surrogate. While displaying different binding affinities to HLA I/HCMV-peptide complexes, all three immunotoxins conferred similar cytotoxic effects on HCMV-peptide loaded cell lines expressing matching HLA I alleles (Fig. 4). Using this experimental setup, we could show that cell lines expressing different HLA I alleles, simulating patients of different HLA status, can be targeted by a TCR-like antibody-immunotoxin repertoire. One major concern of therapeutics that are directed against viral- or tumor-derived peptides presented on HLA class I molecules is the low abundancy of such peptides presented on the cell surface. Peptide loading leads to an abundant display of viral- or tumor-derived peptides on the surface of target cells and may not reflect the biological situation of viral infections or of tumor cells presenting small numbers of altered peptides. To overcome these shortcomings, we obtained the A*0201 positive human lung fibroblast cell line MRC-5. AD169-infected MRC-5 cells were stained with the ETA′-coupled ***C1*** TCR-like antibody. In contrast to primary fibroblasts, staining of HCMV-infected MRC-5 cells with the immunotoxin ***C1-ETA***′ was possible without prior tetramerization. To determine the amount of HCMV-peptide presented on the surface of HCMV-infected MRC-5 cells we performed HCMV-peptide titration experiments and found comparable staining intensities for MRC-5 cells loaded with 10–20 µg/ml HCMV-peptide as for HCMV-infected MRC-5 cells. When incubated with MRC-5 cells that were loaded with 12.5 µg/ml HCMV-peptide, ***C1-ETA***′ still was able to exert cytotoxic effects, demonstrating its ability to be effective even when the target peptide is presented in low concentrations as is the case in HCMV-infection. While very intriguing, the experimental set-up using peptide-loaded cells is still rather artificial and further studies are underway to provide cytotoxicity data of the ***A6-***, ***C7-*** and ***C1-ETA***′ immunotoxins on HCMV-infected cells.

It can only be speculated about the best format, HCMV-specific, TCR-like antibodies could be applied as. The IgG antibody format has well defined pharmacokinetics and pharmacodynamics but is dependent on cellular toxicity in situations where the cellular immune system is suppressed. This could be overcome by incorporating the identified TCR-like Fabs into immunotoxins. With the advent of chimeric antigen receptor (CAR) T cells, another very powerful treatment option for antibodies becomes available [42].

Future studies should focus on extending our approach of a TCR-like antibody repertoire to additional diseases like cancers, which seems particularly promising since the recent discovery of tumor-specific peptides in CLL, AML and CML [43–45].

## Electronic supplementary material

Below is the link to the electronic supplementary material.

Supplementary file1 (PDF 1499 kb)

## Abbreviations

ETA′: Pseudomonas exotoxin A
gMFI: Geometric mean fluorescence intensity
HCMV: Human cytomegalovirus
*k*_a_: Association rate constant
KD: Dissociation constant
*k*_d_: Dissociation rate constant
LCL: Lymphoblastoid cell lines, EBV-transformed B cell
