# Supplementary material for: Characterization of an HLA-restricted and human cytomegalovirus-specific antibody repertoire with therapeutic potential
Source: Cancer Immunol Immunother. 2020 Apr 16;69(8):1535–48. doi: 10.1007/s00262-020-02564-1 (PMC7347513; doi:10.1007/s00262-020-02564-1)
Supplement: Supplementary file 1 — Supplementary file1 (PDF 1499 kb) [file 262_2020_2564_MOESM1_ESM.pdf]

Table S1 More detailed sequence information of the variable regions of all identified HLA/HCMV specific Fab antibodies as gathered from IMGT/V-QUEST. The complete sequences of all 10 Fabs are available from NCBI´s Genbank (see Table S2).

	productive IGH rearranged sequence				productive IGL rearranged sequence		
Fab	V-Gene homology	D-Gene	J-Gene homology	AA Junction	V-Gene homology	J-Gene homology	AA Junction
A*0101							
A6	IGHV1-46*01 / 98,6%	D2-2	J3*02 / 92,0 %	CARNGYCSSTSCYDAFDIW	IGLV2-11*01/94,1%	J3*02 / 86,1%	CCSYAGSSSWVF
F3	IGHV1-46*01/ 99,7%	D1-20	J4*02 / 76,6 %	CASGITGAHDYW	IGLV1-40*02/97,9%	J3*02 / 100%	CQSYDNSLSGPNWWF
A*0201							
A9	IGHV1-18*01 / 95,1%	D1-26	J6*02 / 75,8 %	CARDFGKWDLPMYGMDVW	IGLV1-51*01/92,6%	J1*01 / 91,9%	CGTWNNNLSAYVF
A11	IGHV1-18*01 / 95,8%	D1-26	J6*02 / 77,4 %	CARDFGKWDLPMYGMDVW	IGLV3-21*01/90,3%	J1*01 / 91,7%	CQVWDDRRDHYVF
C1	IGHV1-69*12/ 99,6%	D6-13	J4*02/100%	CARGLAAPDYFDYW	IGLV1-40*02/100%	J1*01/100%	CQSYDSSLSGPFYVF
A*2402							
C12/2	IGHV2-5*02 / 98,3%	D6-13	J4*02 / 97,4%	CARMTYSGSWYSFYYFDYW	IGKV3-11*01 / 93,9%	J2*01 / 91,4%	CQHRRTF
B*0702							
C7	IGHV1-46*01 / 98,3%	D1-14*01	J6*02 / 76,4%	CARYIGIMDVW	IGKV3-20*01 / 97,5 %	J3*01 / 100%	CQQYGSSPLFTF
D10	IGHV3-30*03 / 96,5%	D6-19	J4*02/89,5%	CARARGIGVSGTLYFDFW	IGKV3-20*01 / 98,2 %	J5*01 / 84,2 %	CQQYGSSPGTF
B*0801							
2A2	IGHV1-2*02/97,6%	D4-23*01	JH4*02/79,2%	CAREMGYGGKSEDYW	IGLV3-21*03 / 94,6%	J2or3*01 /86,5%	CQVWDYSSDHVIF
B*3501							
C5	IGHV1-8*02 / 94,1%	D3-3*01	J5*02 / 82,4%	CARQGRLRFLEWYMFDPW	IGKV2-28*01 / 96,9%	J2*01/100%	CMQGLQTPYTF

Table S2 GenBank accession numbers of heavy and light chain variable regions of all identified TCR-like FABs. In the left column the names of selected FAB clones are given and whether the sequence describes the variable heavy (IGHV) or variable light (IGLV) chain.

FAB ID	***GenBank accession numbers***	
A6 IGHV	BankIt2156396 Seq1	MK050824
A6 IGLV	BankIt2156396 Seq2	MK050825
F3 IGHV	BankIt2156396 Seq3	MK050826
F3 IGLV	BankIt2156396 Seq4	MK050827
A9 IGHV	BankIt2156396 Seq5	MK050828
A9 IGLV	BankIt2156396 Seq6	MK050829
A11 IGHV	BankIt2156396 Seq7	MK050830
A11 IGLV	BankIt2156396 Seq8	MK050831
C1 IGHV	BankIt2156396 Seq9	MK050832
C1 IGLV	BankIt2156396 Seq10	MK050833
C12.2 IGHV	BankIt2156396 Seq11	MK050834
C12.2 IGLV	BankIt2156396 Seq12	MK050835
C7 IGHV	BankIt2156396 Seq13	MK050836
C7 IGLV	BankIt2156396 Seq14	MK050837
D10 IGHV	BankIt2156396 Seq15	MK050838
D10 IGLV	BankIt2156396 Seq16	MK050839
2A2 IGHV	BankIt2156396 Seq17	MK050840
2A2 IGLV	BankIt2156396 Seq18	MK050841
C5 IGHV	BankIt2156396 Seq19	MK050842
C5 IGLV	BankIt2156396 Seq20	MK050843

Table S3 Amino acid sequence and originating antigen of the control-peptides used in this study.

	Aa sequence	***Originating antigen***
peptide 1	DTDHYFLRY	CGI-06 protein
peptide 2	VLYDRVLKY	SRP68
peptide 3	KIADRFLLY	LIM domain-only protein 4
peptide 4	KFIDTTSKF	Ribosomal protein L3
peptide 5	TYGEIFEKF	NADH dehydrogenase
peptide 6	IPNEIIHAL	hnRNP M
peptide 7	MPRGVVVTL	E3 ubiquitin-protein ligase HECTD1
peptide 8	NLKLKLHTF	Histone-binding protein RBBP7
peptide 9	RVKGPGISKF	Ectonucleoside triphosphate diphosphohydrolase 1
peptide 10	LPHSSSHWL	Melanocyte protein PMEL
peptide 11	GILGFVFTL	Influenza A matrix protein
peptide 12	SLLMWITQV	NY-ESO-1
peptide 13	TLEEFSAKL	Trypanosoma cruzi KMP-11
peptide 14	ELAGIGILTV	Melan-A

Table S4 Primary human skin fibroblast cell cultures.

ID	***HLA A and B alleles***
Fibro1	A*0201, B*0702
Fibro2	A*0101, A*0201, B*0801, B*3501
Fibro3	A*0301, A*2402, B*0702, B*3801
Fibro4	A*03, A*11, B*07, B*15
Fibro5	A*0201, A*2501, B*0801, B*4001
Fibro6	A*0201, A*0301, B*3501, B*4402
Fibro7	A*0301, A*3303, B*0702, B*3901
Fibro8	A*0101, A*0301, B*0702, B*3503
Fibro9	A*0201, A*0301, B*3501, B*2705
Fibro10	A*0301, A*2402, B*3501, B*5501
Fibro11	A*0101, A*2402, B*1801, B*5701

Table S5 Association and dissociation rate constants and dissociation constants of A6, C1 and C7.

Fabs	ka	kd	KD
A6	$7.78e10^{4}$	$5.89e10^{-4}$	$7.6e10^{-9}$
C1	$4.63e10^{4}$	$2.99e10^{-2}$	$6.6e10^{-7}$
C7	$1.01e10^{4}$	$1.94e10^{-2}$	$1.9e10^{-6}$

a

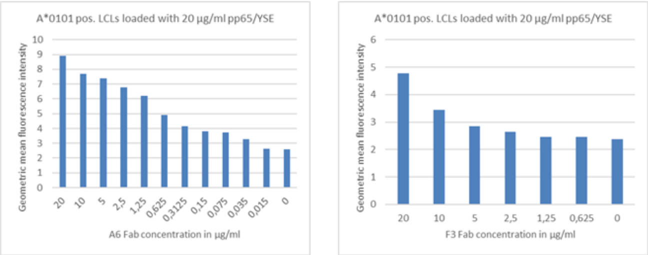
A*0101 pos. LCLs loaded with 20 µg/ml pp65/YSE
Geometric mean fluorescence intensity
A6 Fab concentration in µg/ml
A*0101 pos. LCLs loaded with 20 µg/ml pp65/YSE
Geometric mean fluorescence intensity
F3 Fab concentration in µg/ml

b

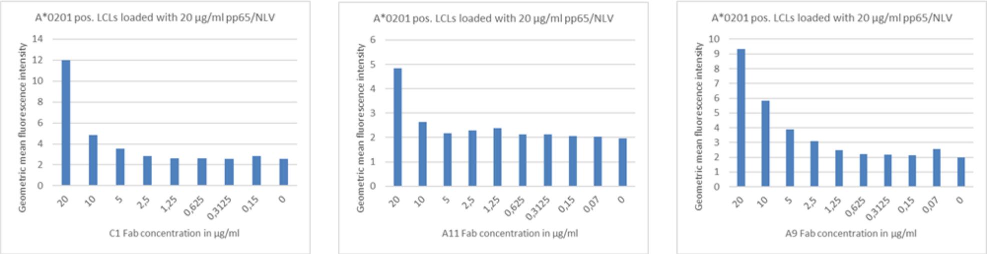
A*0201 pos. LCLs loaded with 20 µg/ml pp65/NLV
Geometric mean fluorescence intensity
C1 Fab concentration in µg/ml
A*0201 pos. LCLs loaded with 20 µg/ml pp65/NLV
Geometric mean fluorescence intensity
A11 Fab concentration in µg/ml
A*0201 pos. LCLs loaded with 20 µg/ml pp65/NLV
Geometric mean fluorescence intensity
A9 Fab concentration in µg/ml

c

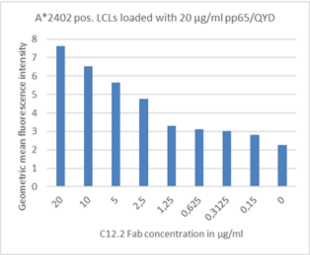
A*2402 pos. LCLs loaded with 20 µg/ml pp65/QYD
Geometric mean fluorescence intensity
C12.2 Fab concentration in µg/ml

d

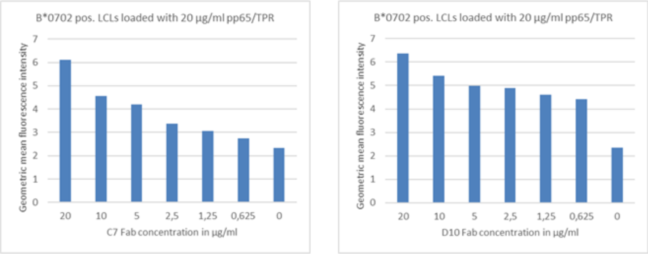
B*0702 pos. LCLs loaded with 20 µg/ml pp65/TPR
Geometric mean fluorescence intensity
C7 Fab concentration in µg/ml
B*0702 pos. LCLs loaded with 20 µg/ml pp65/TPR
Geometric mean fluorescence intensity
D10 Fab concentration in µg/ml

e

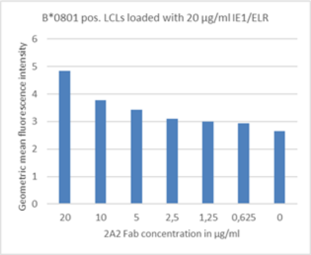
B*0801 pos. LCLs loaded with 20 µg/ml IE1/ELR
Geometric mean fluorescence intensity
2A2 Fab concentration in µg/ml

f

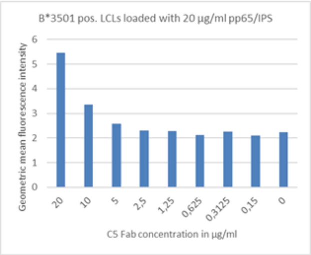
B*3501 pos. LCLs loaded with 20 µg/ml pp65/IPS
Geometric mean fluorescence intensity
C5 Fab concentration in µg/ml

Figure S1 Fab antibody titration.

Titration of the concentration of all 10 Fab antibodies (x-axis) is shown in flow cytometry staining experiments of HCMV-peptide loaded LCLs and measured by the geometric mean fluorescence intensity (y-axis). LCLs of different HLA alleles were constantly loaded with 20 µg/ml of their respective HCMV-peptide. HCMV-peptide-loaded LCLs were then stained with HLA-matching, HCMV-specific Fab antibodies in decreasing concentration from 20 µg/ml to 0 µg/ml. **a)** displays the HLA A*0101 restricted, HCMV-specific Fab clones A6 and F3 in diluted concentrations on A*0101 positive LCLs loaded with the HCMV-peptide YSE (derived from pp65). Binding of A6 can be detected at concentrations of < 1 µg/ml whereas F3 starts to show binding to HCMV-peptide loaded LCLs at concentrations of 10 µg/ml. In **b)** the A*0201 restricted HCMV Fab clones C1, A11 and A9 are tested for binding to HLA A*0201 positive LCLs (loaded with 20 µg/ml of the pp65-derived HCMV peptide NLV) in different concentrations. A9, C1 and A11 begin to bind to HLA-matching LCLs at concentrations of 5 µg/l, 10 µg/l and 20 µg/ml, respectively. **c)** illustrates corresponding Fab titrations for the clone C12.2 (HLA*A2402 restricted, LCLs pulsed with QYD of pp65). C12.2 concentrations can be halved down to 2.5 µg/ml while still showing binding to LCLs. **d)** C7 and D10 concentrations (both HLA*B0702 restricted, LCLs pulsed with TPR of pp65) can be diluted to 5 and 0.625 µg/ml maintaining binding capacity to HCMV-peptide pulsed LCLs. **e) and f)** 2A2 (HLA*B0801 restricted, LCLs pulsed with ELR of IE1) and C5 (HLA*B3501 restricted, LCLs pulsed with IPS of pp65) demonstrate relevant binding to peptide-pulsed LCLs only at 20 µg/ml.

a

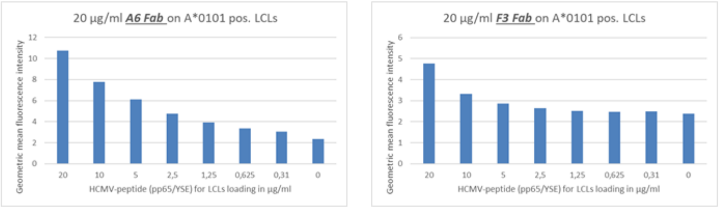

20 µg/ml A6 Fab on A*0101 pos. LCLs
Geometric mean fluorescence intensity
HCMV-peptide (pp65/YSE) for LCLs loading in µg/ml
20 µg/ml F3 Fab on A*0101 pos. LCLs
Geometric mean fluorescence intensity
HCMV-peptide (pp65/YSE) for LCLs loading in µg/ml

b

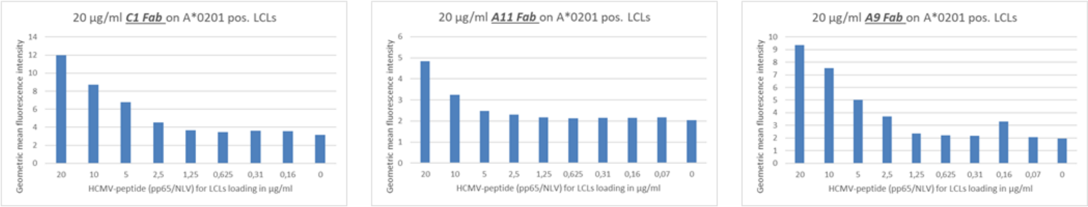

20 µg/ml C1 Fab on A*0201 pos. LCLs
Geometric mean fluorescence intensity
HCMV-peptide (pp65/NLV) for LCLs loading in µg/ml
20 µg/ml A11 Fab on A*0201 pos. LCLs
Geometric mean fluorescence intensity
HCMV-peptide (pp65/NLV) for LCLs loading in µg/ml
20 µg/ml A9 Fab on A*0201 pos. LCLs
Geometric mean fluorescence intensity
HCMV-peptide (pp65/NLV) for LCLs loading in µg/ml

c

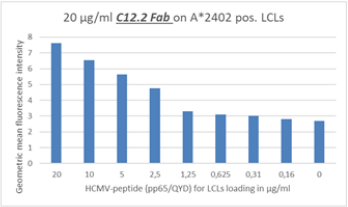

20 µg/ml C12.2 Fab on A*2402 pos. LCLs
Geometric mean fluorescence intensity
HCMV-peptide (pp65/QYD) for LCLs loading in µg/ml

d

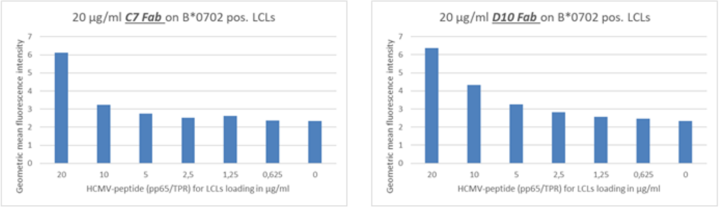

20 µg/ml C7 Fab on B*0702 pos. LCLs
Geometric mean fluorescence intensity
HCMV-peptide (pp65/TPR) for LCLs loading in µg/ml
20 µg/ml D10 Fab on B*0702 pos. LCLs
Geometric mean fluorescence intensity
HCMV-peptide (pp65/TPR) for LCLs loading in µg/ml

e

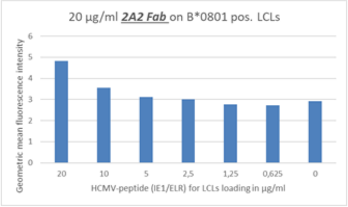

20 µg/ml 2A2 Fab on B*0801 pos. LCLs
Geometric mean fluorescence intensity
HCMV-peptide (IE1/ELR) for LCLs loading in µg/ml

f

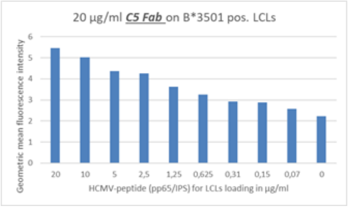

20 µg/ml C5 Fab on B*3501 pos. LCLs
Geometric mean fluorescence intensity
HCMV-peptide (pp65/IPS) for LCLs loading in µg/ml

Figure S2 HCMV-peptide titration.
Flow cytometry with assessment of the geometric mean fluorescence intensity (y-axis) was used for HCMV-peptide titration experiments. Concentrations of Fab clones used for LCL staining were held constant at 20 µg/ml. LCLs expressing different HLA I alleles were loaded with corresponding HCMV-peptides (Table 1) at decreasing concentrations starting from 20 µg/ml (x-axis). **a)** shows the binding intensity of the A*0101 restricted, HCMV specific Fabs A6 and F3 to LCLs pulsed with decreasing concentrations of the pp65-derived peptide YSE. For A6, peptide loading of LCLs with 2,5 µg/ml YSE seems to be sufficient to show binding whereas for F3, HCMV-peptide pulsing with more than 10 µg/ml is required to show it´s binding to LCLs. **b)** Down to 5 µg/ml of the pp65-derived HCMV-peptide NLV is needed for peptide-pulsing in order to show binding capacity of the A*0201 restricted, HCMV specific Fabs C1 and A9 to A*0201 expressing LCLs. When staining with A11, peptide-pulsing of LCLs with more than 10 µg/ml of NLV is required to detect binding. **c)** C12.2 Fab staining of A*2402 positive LCLs loaded with different concentrations of the HCMV-peptide QYD (derived from pp65). **d)** C7 and D10 Fab staining of B*0702 expressing LCLs loaded with the pp65-derived HCMV-peptide TPR. For positive LCL staining with the Fabs C7 and D10 HCMV-peptide concentrations of >10 µg/ml and 5 – 10 µg/ml, respectively, are required. **e)** 2A2 Fab staining (20 µg/ml) of B*0801 positive LCLs loaded with different concentrations of the HCMV-peptide ELR (derived from IE1) is illustrated. **f)** B*3501 positive LCLs can be stained with the Fab clone C5 when LCLs are loaded with the HCMV-peptide IPS (derived from pp65) starting at peptide concentrations of approximately 2.5 µg/ml.

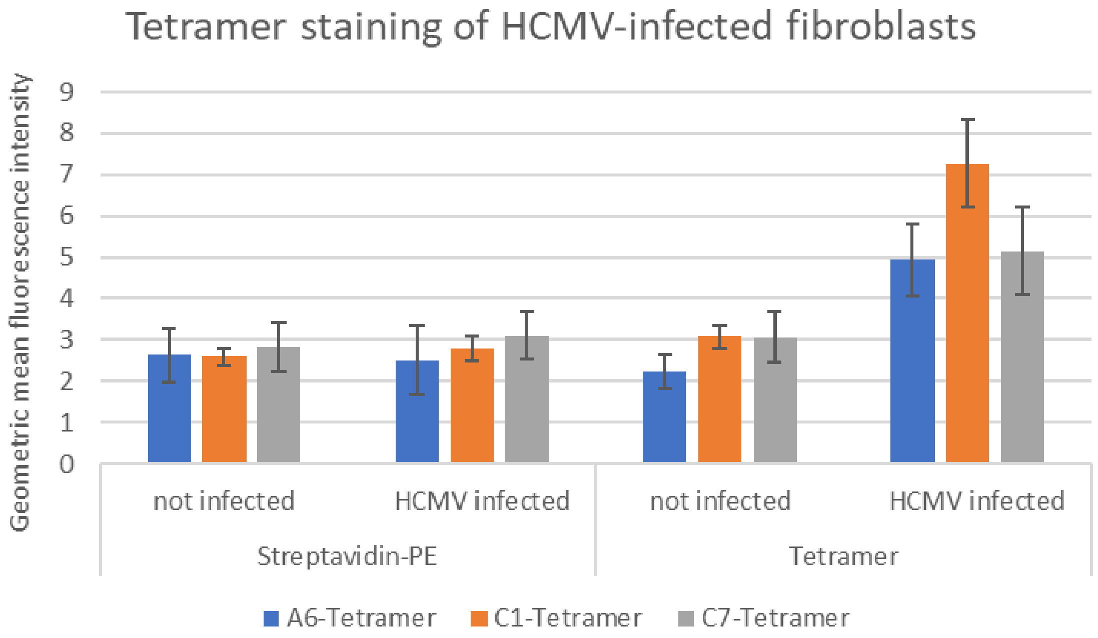

Figure S3 Tetramer staining of HCMV-infected primary fibroblasts.
HCMV-infected fibroblasts expressing different HLA alleles were incubated with tetramers of the HLA/HCMV-specific Fabs A6 (A*0101 restricted), C1 (A*0201 restricted) and C7 (B*0702 restricted). Staining intensity as assessed by flow cytometry was measured using the geometric mean fluorescence intensity which is plotted on the y-axis. Staining experiments were performed 3 – 5 days after HCMV infection. Blue columns show the mean of 4 technical repeats of staining experiments with the A6-tetramer on HCMV-infected cells of Fibro2 and Fibro11. Orange columns represent the mean of 14 C1-tetramer staining experiments on cells of Fibro2, Fibro5, Fibro6, Fibro9 and MRC-5. Grey columns show the mean of 6 repeats of the C7-tetramer on Fibro4 and Fibro7. Bars indicate standard errors. As control, tetramer staining was performed on uninfected cells and with streptavidin-PE alone.

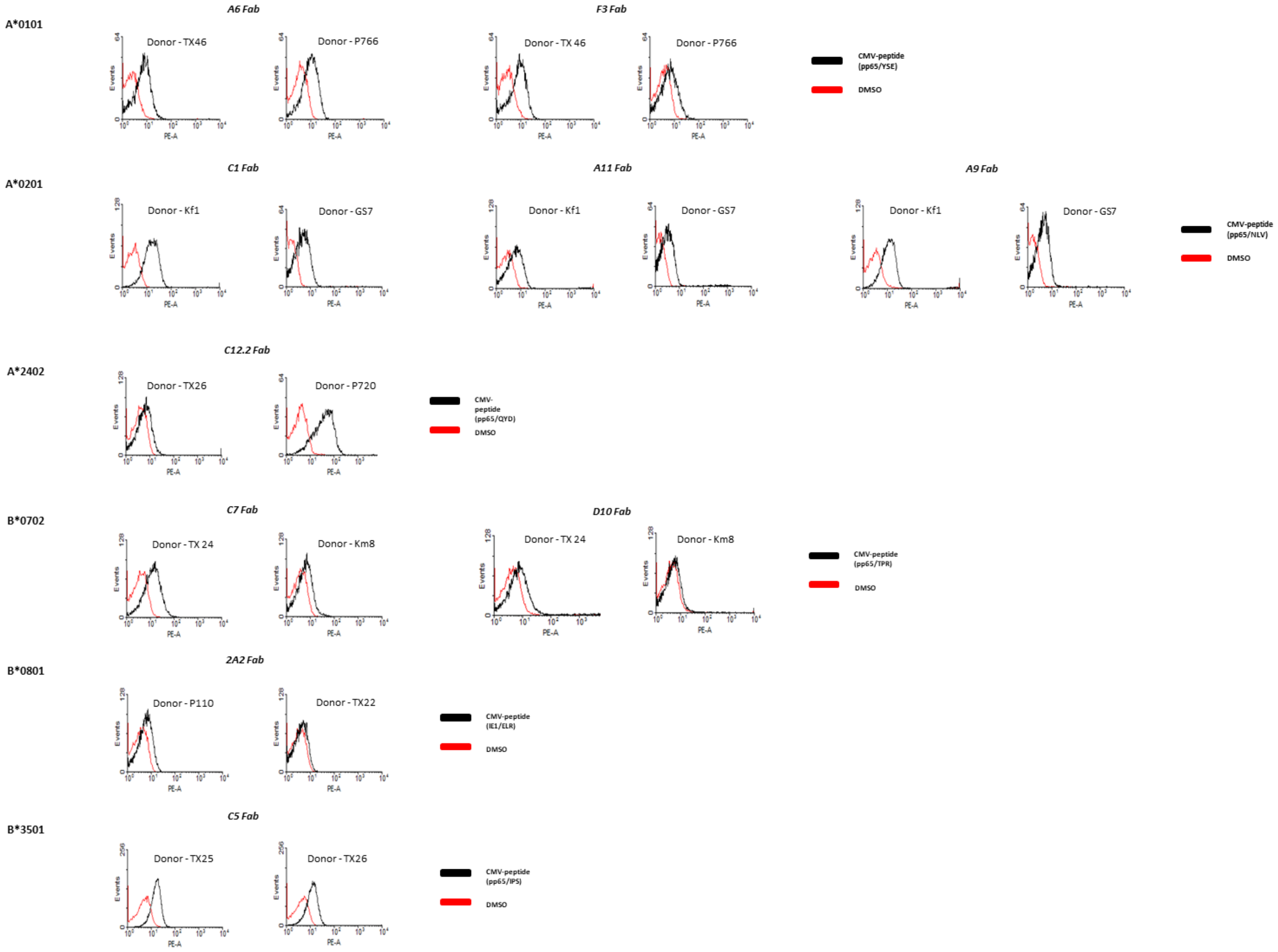

Figure S4 Interpatient variability.
In order to show the ability of our TCR-like, HCMV-specific Fab antibodies to bind to the lymphocytes of different donors of the same HLA I type, i.e. to exclude relevant interpatient variability, we loaded peripheral blood of 2 donors expressing the same HLA I allele with HCMV peptides (20 µg/ml) and tested all identified TCR-like, HCMV-specific Fabs (50µg/ml) for binding by flow cytometry. All Fabs are assorted by their HLA I restriction and respective HCMV-peptides used for lymphocyte-pulsing are given as 3-letter code (see table S3). In summary, all selected HCMV-specific TCR-like Fabs showed binding to HCMV-peptide loaded lymphocytes of different donors expressing the same HLA I allele. For most Fabs, some difference in binding affinity was detected, but their general ability to bind to HCMV-peptide-loaded lymphocytes of matching HLA I-status was maintained.

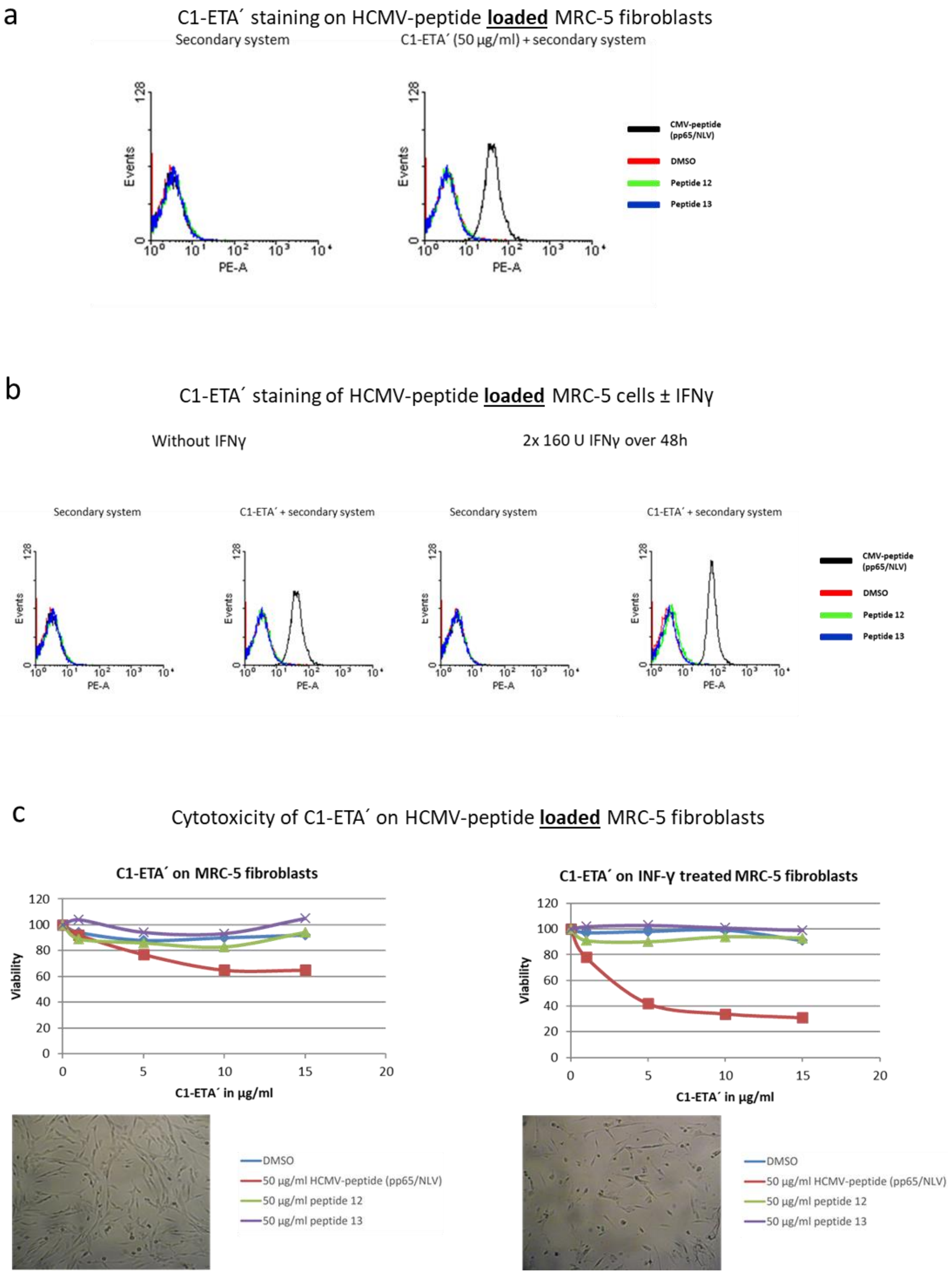

a
C1-ETA´ staining on HCMV-peptide loaded MRC-5 fibroblasts
Secondary system
C1-ETA´ (50 µg/ml) + secondary system
Events
PE-A
CMV-peptide (pp65/NLV)
DMSO
Peptide 12
Peptide 13
b
C1-ETA´ staining of HCMV-peptide loaded MRC-5 cells ± IFNγ
Without IFNγ
2x 160 U IFNγ over 48h
Secondary system
C1-ETA´ + secondary system
Secondary system
C1-ETA´ + secondary system
CMV-peptide (pp65/NLV)
DMSO
Peptide 12
Peptide 13
c
Cytotoxicity of C1-ETA´ on HCMV-peptide loaded MRC-5 fibroblasts
C1-ETA´ on MRC-5 fibroblasts
Viability
C1-ETA´ in µg/ml
C1-ETA´ on INF-γ treated MRC-5 fibroblasts
Viability
C1-ETA´ in µg/ml
DMSO
50 µg/ml HCMV-peptide (pp65/NLV)
50 µg/ml peptide 12
50 µg/ml peptide 13
DMSO
50 µg/ml HCMV-peptide (pp65/NLV)
50 µg/ml peptide 12
50 µg/ml peptide 13

Figure S5 Effects of Interferon gamma (IFNγ) on HCMV-peptide loaded MRC-5 cells. MRC-5 fibroblasts can be infected with HCMV similar to primary fibroblasts and express the HLA I allele A*0201. The HLA A*0201-restricted, HCMV-specific Fab antibody C1, coupled to *Pseudomonas* Exotoxin A (C1-ETA´), was tested for binding to HCMV-peptide loaded MRC-5 cells. For staining experiments MRC-5 cells were pulsed with the pp65-derived HCMV-peptide NLV (see table 1) at 50 µg/ml. For cytotoxicity assays of C1-ETA´, MRC-5 fibroblasts were loaded with 50 µg/ml HCMV-peptide NLV. **(a)** When loaded with 50 µg/ml HCMV-peptide (pp65/NLV), MRC-5 cells can be specifically stained with the HLA A*0201-restricted, HCMV-specific Fab antibody C1, that is coupled to *Pseudomonas* Exotoxin A. When loaded with control peptides (Table S3) C1-ETA´ does not bind to MRC-5 cells. **(b)** Stimulation of MRC-5 fibroblasts with Interferon gamma (2 x 160 U over 48h) did only minimally improve the affinity of C1-ETA´ to HCMV-peptide loaded MRC-5 cells. **(c)** For cytotoxicity assays, MRC-5 fibroblasts were either loaded with 50 µg/ml HCMV-peptide (pp65/NLV) or the same amount of control peptides and subsequently incubated with C1-ETA´ in increasing concentration from 1 µg/ml to 15 µg/ml. Viability was assessed using alamarBlue™ as described in material and methods. Without the addition of Interferon gamma (2 x 160 U in 48h) the cytotoxic effects of C1-ETA´ on HCMV-peptide loaded MRC-5 fibroblasts is weak. After adding Interferon gamma 2 times over 48 hours prior to cytotoxicity assays, C1-ETA´ shows potent cytotoxic effects against HCMV-peptide pulsed MRC-5 cells. We speculate that Interferon gamma leads to increased internalization of HLA complexes since binding of C1-ETA´ to HCMV-peptide loaded MRC-5 fibroblasts is not influenced by Interferon gamma as shown in (B).

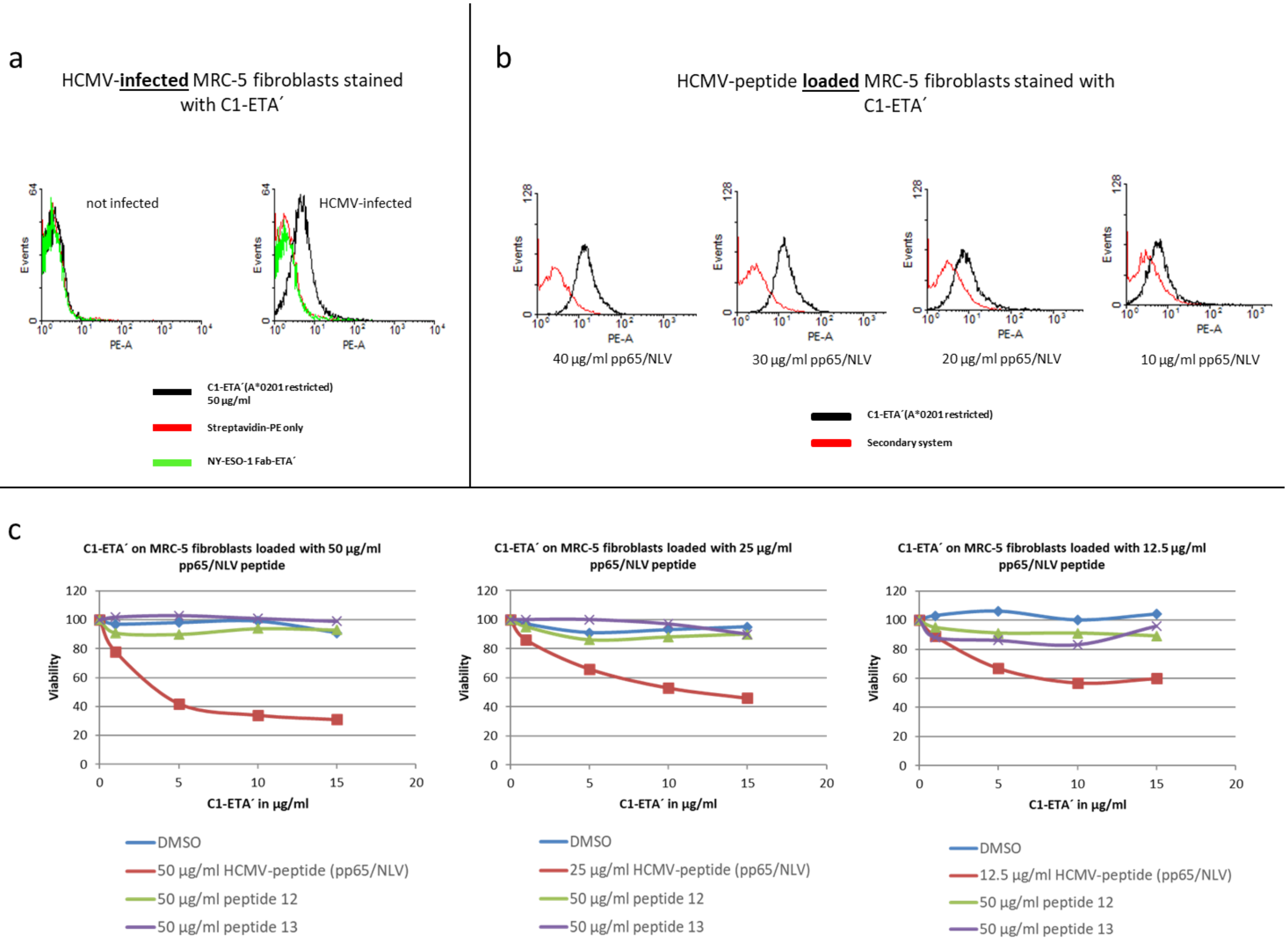

Figure S6 MRC-5 fibroblast cell line experiments.
In figure 4, we demonstrate the ability of 3 different ETA´ conjugated HLA I-restricted and HCMV-specific Fabs to kill HCMV-peptide loaded cell lines expressing different HLA I alleles providing proof of concept results for respective Fabs as therapeutic options in the treatment of HCMV infected cells. In order to generate a more realistic test setting we used the fibroblast cell line MRC-5. After infection with the HCMV strain AD169 (MOI 0.5-1.0), we were able to show binding of the ETA´-conjugated, A*0201 restricted and HCMV specific Fab antibody C1 specifically to infected MRC-5 cells and not to uninfected MRC-5 cells **(a)**. To determine the amount of HCMV-peptide presented on the surface of HCMV-infected MRC-5 cells we performed HCMV-peptide titration experiments and found comparable staining intensities for MRC-5 cells loaded with 10 – 20 µg/ml HCMV-peptide as for HCMV-infected MRC-5 cells **(b)**. When incubated with MRC-5 cells that were loaded with HCMV-peptide at 12.5 µg/ml, C1-ETA´ still was able to exert cytotoxic effects, demonstrating its ability to be effective even when the target peptide is presented only in low concentrations mimicking HCMV-infection **(c)**. When loaded with higher HCMV-peptide concentrations the cytotoxic effects of C1-ETA´ on MRC-5 cells increase **(c)**.

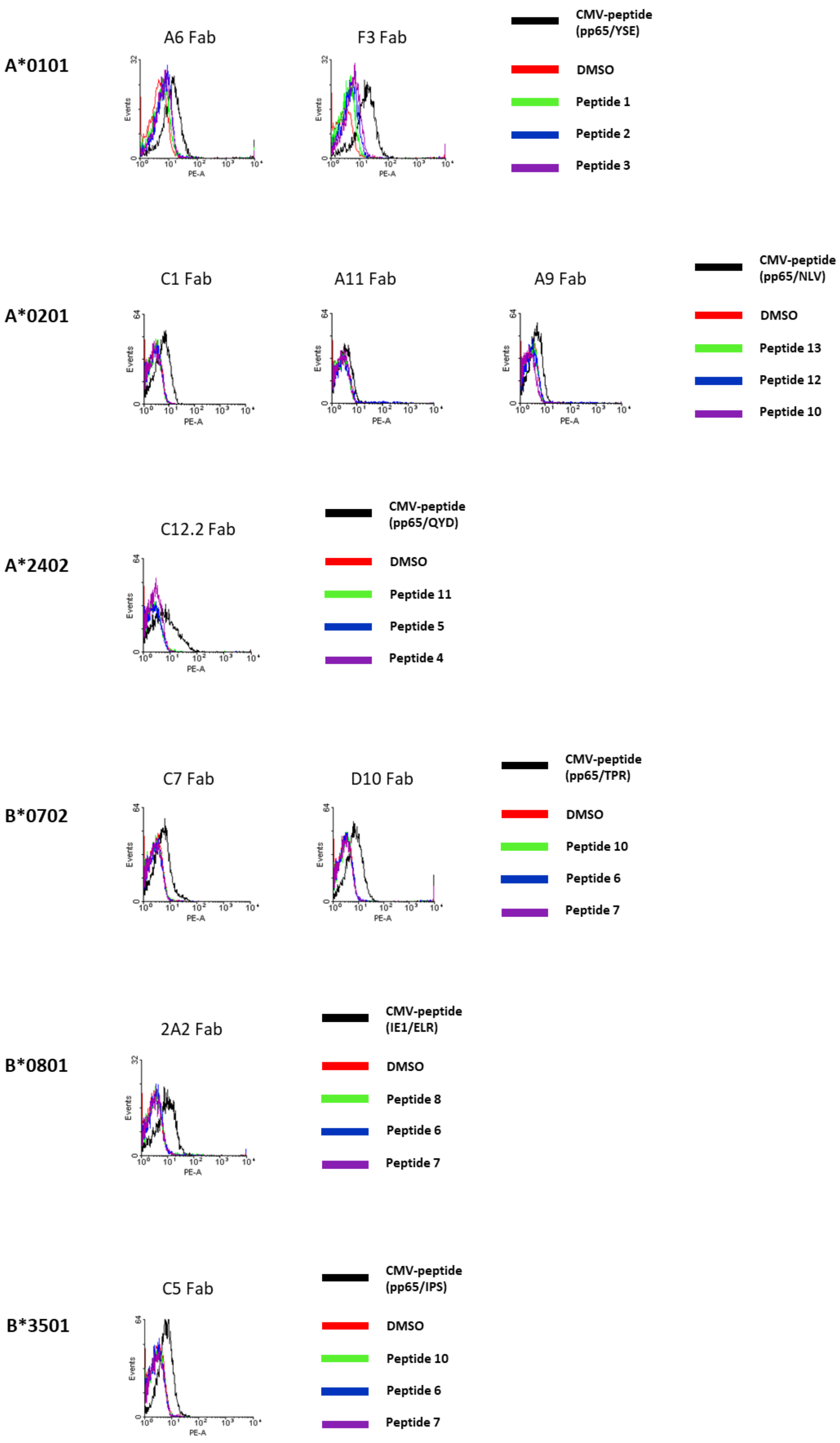
A*0101
A6 Fab
F3 Fab
CMV-peptide (pp65/YSE)
DMSO
Peptide 1
Peptide 2
Peptide 3
A*0201
C1 Fab
A11 Fab
A9 Fab
CMV-peptide (pp65/NLV)
DMSO
Peptide 13
Peptide 12
Peptide 10
A*2402
C12.2 Fab
CMV-peptide (pp65/QYD)
DMSO
Peptide 11
Peptide 5
Peptide 4
B*0702
C7 Fab
D10 Fab
CMV-peptide (pp65/TPR)
DMSO
Peptide 10
Peptide 6
Peptide 7
B*0801
2A2 Fab
CMV-peptide (IE1/ELR)
DMSO
Peptide 8
Peptide 6
Peptide 7
B*3501
C5 Fab
CMV-peptide (pp65/IPS)
DMSO
Peptide 10
Peptide 6
Peptide 7
Events
PE-A

Figure S7 Binding assay of HLA I/HCMV-peptide-specific Fabs on lymphocytes.
EDTA blood from HLA-typed donors was pulsed with HCMV-peptides and three control-peptides after erythrocyte lysis. Staining experiments were done as described with LCLs using 20 µg/ml HCMV-peptides for lymphocyte pulsing and 50 µg/ml HLA I/HCMV-peptide-specific Fabs. Data analysis was done on gated lymphocytes. Histograms are assorted according to histograms in Figure 2 by HLA alleles. All HLA I/HCMV-peptide-specific Fab antibodies that showed binding to HCMV-peptide pulsed LCLs also bound to HCMV-peptide pulsed blood lymphocytes. Control-peptides used were the same as with LCLs in Figure 2. As compared to HCMV-peptide loaded pure LCLs, binding capacity to lymphocytes after staining of whole EDTA blood was weaker as measured by flowcytometry.

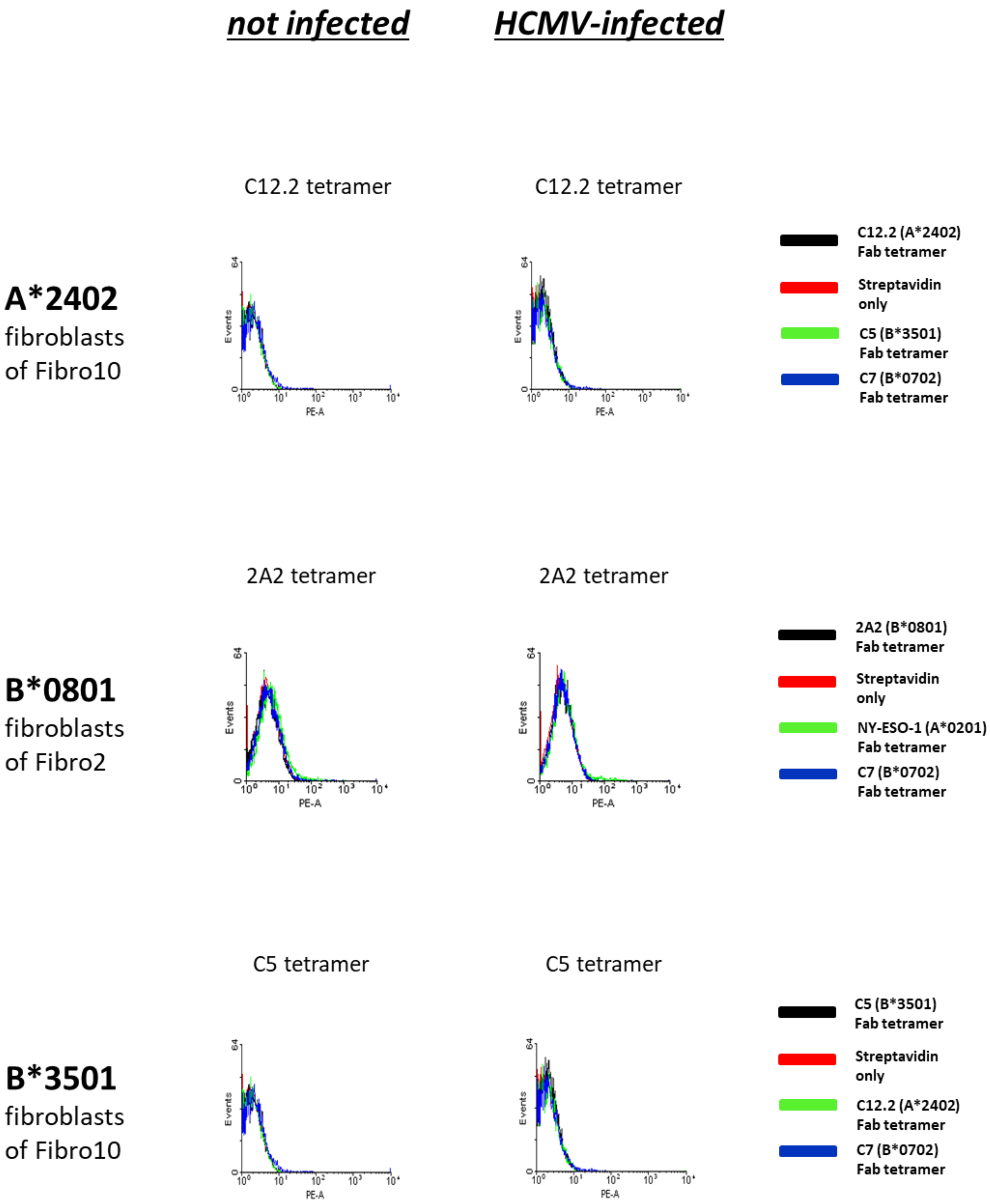

not infected
HCMV-infected
A*2402
fibroblasts of Fibro10
C12.2 tetramer
C12.2 tetramer
C12.2 (A*2402) Fab tetramer
Streptavidin only
C5 (B*3501) Fab tetramer
C7 (B*0702) Fab tetramer
B*0801
fibroblasts of Fibro2
2A2 tetramer
2A2 tetramer
2A2 (B*0801) Fab tetramer
Streptavidin only
NY-ESO-1 (A*0201) Fab tetramer
C7 (B*0702) Fab tetramer
B*3501
fibroblasts of Fibro10
C5 tetramer
C5 tetramer
C5 (B*3501) Fab tetramer
Streptavidin only
C12.2 (A*2402) Fab tetramer
C7 (B*0702) Fab tetramer
Events
PE-A

Figure S8 Negative binding assays of HLA I/HCMV-peptide-specific Fab tetramers to HCMV-infected fibroblasts.

The HCMV-specific tetramerized Fabs C12.2, 2A2 and C5 that are restricted to the HLA alleles A*2402, B*0801 and B*3501 showed no binding to either infected or uninfected fibroblasts with permissive HLA alleles. C12.2 (A*2402) was tested on Fibro10 fibroblasts, 2A2 (B*0801) was incubated with cells of Fibro2 and Fibro5 cell cultures and C5 (B*3501) was tested on cells of the primary human skin fibroblast culture Fibro10. Different HCMV-specific, HLA I restricted tetramerized Fabs and the tetramerized NY-ESO-1 specific, HLA A*0201 restricted Fab were used as controls (depicted and assorted by color on the right side).

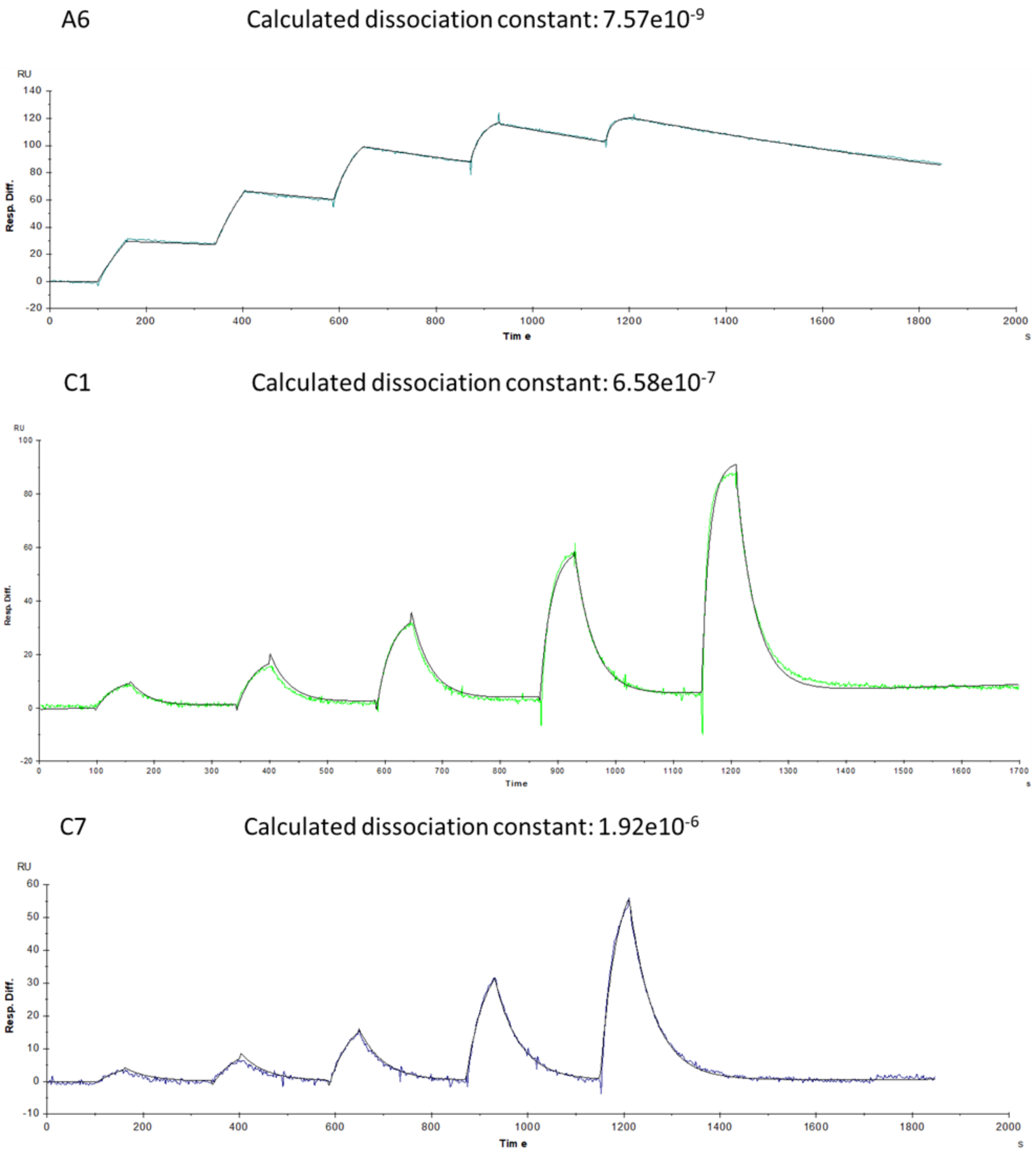

Figure S9 Surface plasmon resonance imaging for affinity calculation of HCMV specific, TCR-like Fab antibodies.

Biotinylated monomeric HLA-I/HCMV-peptide complexes (ligand) were immobilized on a streptavidin-coupled CM5 Chip. Fab antibodies (analyte) were injected in concentrations of 1.0 µM, 0.5 µM, 0.25 µM, 0.125 µM and 0.0625 µM. The Fab antibodies A6, C1 and C7 were injected sequentially without regeneration using a single-cycle kinetics protocol. The HLA A*0101 restricted, HCMV specific Fab antibody A6 showed a dissociation constant (KD) of $7.57e10^{-9}$. For C1 a KD value of $6.58e10^{-7}$ was calculated. C7, the HLA B*0702 restricted, HCMV specific Fab antibody, showed the lowest affinity with a dissociation constant of $1.92e10^{-6}$.
